# A proteomics-based study on the mechanisms of *Terminalia chebula* Retz processed *Aconitum kusnezoffii* Reichb against rheumatoid arthritis and its cardiotoxicity reduction

**DOI:** 10.1186/s13020-025-01306-8

**Published:** 2026-01-09

**Authors:** Jia-hua Wang, Yun-lu Liu, Chen-chen Zhang, Lin Song, Li Mei, Ri-na Sa, Ya Gao, Ya Tu

**Affiliations:** 1 Experimental Research Center, China Academy of Chinese Medical Sciences, No. 16 South Xiaojie, Dongzhimennei, Dongcheng District, Beijing, 100700 China; 2 Inner Mongolia Medical University, Hohhot, 010110 China; 3 Anhui Institute of Food and Drug Control, Drug Inspection and Research Institute, The intersection of Baohe Avenue and Urumqi Road, Baohe District, Hefei, 230051 China

**Keywords:** *Hezi*-processed *Caowu*, Rheumatoid arthritis, Cardiotoxicity, Proteomics, Molecular docking

## Abstract

**Background:**

Although the efficacy of *Aconitum kusnezoffii* Reichb (*Caowu*) in treating rheumatoid arthritis is well-established, its severe toxic side effects have attracted widespread attention from researchers both domestically and internationally. Processing is an important method for reducing the toxicity of *Caowu* and ensuring its safe clinical application, with the processing of *Caowu* using the *Terminalia chebula* Retz (*Hezi*) decoction method being a distinctive approach in Mongolian medicine. The majority of current studies on HC are still conducted in animal models under normal physiological conditions, failing to adequately account for the impact of pathological states on its efficacy and toxicity. Moreover, the material basis underlying its effects and the molecular mechanisms through which processing reduces toxicity while preserving therapeutic efficacy remain unclear.

**Purpose:**

This study employs proteomics to uncover changes in toxicity- and efficacy-related proteins during the in vivo action of Hezi Processed Caowu (HC), thereby exploring the molecular mechanisms behind its ‘‘toxicity reduction while efficacy retention’’.

**Methods:**

Using LC–MS/MS technology, based on the Collagen- Induced Arthritis (CIA) rat model, we combined in vivo and in vitro chemical composition analysis, in vivo pharmacology/toxicology, proteomics, molecular docking, and other research methods to explore its molecular mechanism.

**Results:**

Using UPLC-Q-Orbitrap-MS/MS technology, 43 compounds were identified in the positive ion mode for HC. After administration of HC, 24 parent compounds were detected in plasma and 25 parent components were detected in the heart. A CIA rat model was established to evaluate the anti-RA (Rheumatoid Arthritis) pharmacological effects of HC. It was found that HC could reduce foot swelling in CIA rats, lower the arthritis index, and decrease the secretion of MMP-2, MMP-3, TNF-α, and IL-6. Proteomic analysis revealed that, compared with the CIA group, administration of HC significantly downregulated the protein expression of Ctsk, Acp5, and Casp3. Molecular docking was employed to simulate the spatial conformation between the core differentially expressed target proteins and the blood-absorbed bioactive components of the HC. The highest docking scores were observed between Ctsk and benzoylaconitine, Casp3 and aconitine, as well as Acp5 and ellagic acid. After long-term treatment of CIA rats with raw *Caowu* (RC) and HC, histopathological examination and electrocardiogram (ECG) detection indicated significant cardiotoxicity in the RC group, which was ameliorated by HC group. Subsequent biochemical analysis showed that, compared to the raw aconite group, the levels of AST, ALP, LDH, CK, CK-MB, TP, and TBA were reduced in the HC group. Proteomic studies further demonstrated that the expression of Kng1 and Sod1 was downregulated in the HC group compared to the RC group. Western blot analysis confirmed that Nrf2, Kng1, and Sod1 were highly expressed in the RC group, whereas their expression was reduced in HC group. Additionally, compared with the RC group, HC decreased the levels of Casp3 and Bax, while increasing the expression of Bcl2. Further analysis using molecular docking technology validated the spatial conformation of differentially expressed core target proteins with cardiac active components. Among these, Nrf2 had the highest docking score with benzoylmesaconine, Kng1 had the highest docking score with chasmanine, and Sod1 had the highest docking score with benzoylaconitine. Casp3 had the highest docking score with aconitine, Bax had the highest docking score with senbusine A, and Bcl2 had the highest docking score with mesaconine.

**Conclusions:**

HC exerts its anti-RA effects by prolonging the retention time of anti-inflammatory components in the body, reducing the expression of Ctsk, Acp5, and Casp3 proteins, and inhibiting bone erosion and joint damage; it also reduces cardiac toxicity by reducing oxidative stress and protecting against apoptosis, thereby forming a multidimensional detoxification mechanism. This study focused on the anti-inflammatory activity of HC, integrating blood component analysis, cardiac tissue distribution detection, and disease model-driven synovial and cardiac proteomics analysis to scientifically elucidate the detoxification and efficacy principles of HC, providing new strategies for comprehensive research on detoxification and efficacy in the preparation of toxic herbs for ethnic minorities.

**Supplementary Information:**

The online version contains supplementary material available at 10.1186/s13020-025-01306-8.

## Introduction

Traditional Chinese medicine (TCM) has a long history and is widely used worldwide for disease treatment and prevention. However, adverse reaction events occasionally occur, and safety concerns regarding TCM have gradually gained attention, with its potential toxic side effects not to be overlooked [1, 2]. The occurrence of toxic reactions from TCM is not only related to the nature of the drugs themselves but is also closely associated with the physiological state of the body during administration [3]. Therefore, research on the toxicity of TCM urgently needs to be grounded in the physiological state background that reflects the characteristics of TCM application and the actual occurrence of toxicity, and to deeply explore the intrinsic connection between toxicity occurrence and physiological state. This research paradigm is not only crucial to the construction and improvement of the toxicity theory system of TCM, but also can provide the core scientific basis and practical guidance for clinical medication [4].

Raw *Aconitum kusnezoffii* Reichb (*Caowu*), the dried root tuber of the plant *Aconitum kusnezoffii* Reichb, is warm in nature and pungent in taste. It has anti-inflammatory and analgesic effects and can be used to treat rheumatoid arthritis [2, 5]. According to Mongolian medical theory, it is highly toxic, particularly to the heart, so it must be processed to reduce its toxicity before it can be used clinically. Mongolian medicine's characteristic processing method includes *Terminalia chebula* Retz (*Hezi*) decoction processing, whose principle lies in the tannins in Hezi combining with the alkaloids in *Caowu* to form insoluble salts, thereby reducing the content of highly toxic diester-type alkaloids while increasing the proportion of lipid-type alkaloids [6]. Although previous studies have analyzed the intestinal absorption, intestinal bacterial metabolism, and hepatic metabolic components of *Caowu* before and after processing [7], current research on Hezi Processed *Caowu* (HC) has primarily focused on its toxicity-reducing effects in isolation, lacking a comprehensive evaluation within the broader context of its anti-inflammatory and analgesic effects. More importantly, the key material basis for its pharmacological effects and toxicity, as well as the multi-target molecular mechanisms underlying its “toxicity reduction while preserving efficacy,” have yet to be systematically elucidated.

In the study of molecular mechanisms, proteomics has gradually come into focus. It investigates differences in protein expression levels, modification states, interactions, abundance, and characteristics during disease progression and drug treatment [8]. This methodology integrates gel-based and chromatographic separation techniques with mass spectrometry (MS) analysis, and bioinformatics methods. It not only enables in-depth analysis of individual proteins but also possesses powerful capabilities for processing complex protein samples, effectively addressing complex biological questions in medical and basic science fields, thereby revealing and exploring biological processes and their related pathways in detail [9, 10]. This characteristic makes it particularly suitable for analyzing the complexity of traditional Chinese medicine (TCM). Due to the diverse sources of TCM herbs, their specific proteins may serve as both core bioactive components and biomarkers of herbal origin [11]. Additionally, TCM formulas consist of multiple herbs containing a wide range of chemical components, and individual compounds often act on multiple protein targets, exhibiting a “multi-component, multi-target” characteristic. Therefore, when faced with the highly complex “multi-component, multi-target” system of TCM, proteomics, with its systematic analytical capabilities, has become the most powerful tool for revealing the key functional proteins of TCM and their underlying pharmacological mechanisms.

This study focuses on Mongolian medicine HC, aiming to systematically elucidate its molecular mechanisms underlying “toxicity reduction while maintaining efficacy.” We first characterized blood-borne components of HC and their cardiac distribution profiles using UHPLC-Q-TOF/MS technology. Subsequently, through the collagen-induced arthritis (CIA) model combined with proteomic analysis, we identified key therapeutic targets responsible for HC's anti-rheumatoid arthritis (RA) effects, thereby clarifying the immune-regulatory network essential for its efficacy preservation. Further investigation involving detection of cardiotoxicity biomarkers and myocardial proteomics revealed specifically regulated detoxification pathways. Ultimately, integration of these multidimensional datasets enabled the construction of a comprehensive ‘‘chemical constituents-pharmacological effects/toxicity manifestations’’ correlation model.

## Materials and methods

### Reagents and instruments

The Mongolian medicinal herb *Caowu* is harvested from the Hanshan region of Tongliao City, Inner Mongolia, and has been identified by Professor Buhebate of Inner Mongolia University of Nationalities as the dried tuberous root of *Aconitum kusnezoffii* Reichb. Hezi was purchased from Jinan Renhe Traditional Chinese Medicine Decoction Pieces Co., Ltd., batch number 20140101. It was identified by Professor Song Lin of the Mongolian Medicine College of Inner Mongolia Medical University as the dried mature fruit of *Terminalia chebula* Retz. IL-1β ELISA kit was purchased from Wuhan EIAab Biotechnology Co., LTD. TNF-α ELISA kit and IL-6 ELISA kit were purchased from Wuhan Huamei Biological Engineering Co., LTD. H&E staining solution and paraffin embedding box were purchased from Wuhan Servicebio Biotechnology Co., LTD. Nrf2 antibody (16,396-1-AP), SOD1 antibody (67,480-1-lg), Beta actin antibody (66,009-1-lg), goat anti-rabbit antibody (B900210) and goat anti-mouse antibody (RGAM001) were purchased from Wuhan Sanying Biotechnology Co., LTD. Caspase 3 antibody (A0214), Bax antibody (A7626), and Bcl2 antibody (A20777) were purchased from Wuhan ABclonal Biotechnology Co., LTD. Total bile acids (TBA) Assay Kit, alkaline phosphatase (ALP) Assay Kit, alanine transaminase (ALT) Assay Kit, aspartate transaminase (AST) Assay Kit, creatine kinase (CK) Assay Kit, lactate dehydrogenase (LDH) Assay Kit, and total protein (TP) Assay Kit were purchased from Beijing Beijian Xinchuangyuan Biotechnology Co., Ltd.

The liquid chromatography-mass spectrometry instrument was purchased from Thermo Fisher, USA. ZOX Extend-C18 column was purchased from Agilent Technologies (USA). The pathological microtome was purchased from Leica Instruments (Shanghai) Co., LTD., and the Pannoramic panoramic slice scanner was purchased from 3DHIESTECH (Hungary).

### Preparation of medicinal materials

First, we take Hezi, with a dosage of half that of Raw *Caowu* (RC). We boil it for 1 h using 10 times its volume of water. Then, we filter it through 4 layers of gauze. Next, we soak RC in the filtrate for 2 days, and then boil it for another 8 h. After that, we remove and place on a square tray to dry at 60 °C to obtain HC. When used clinically, it was diluted with 0.5%CMC-Na solution and set aside.

### Animals

SPF-grade male SD rats, weighing 230–260 g, were provided by Beijing Vital River Laboratory Animal Technology Co., Ltd. The animal procedures were approved by the ethics committee with the approval number ERCCACMS21-2405-04. The rats were housed in the Institute of Basic Theory of Traditional Chinese Medicine, China Academy of Chinese Medical Sciences, with the temperature maintained at 23–25 ℃, humidity at 40–60%.

Under ice-bath conditions, the bovine type II collagen acetic acid solution (2 mg/mL) and incomplete Freund's adjuvant (1 mg∙mL^−1^) were mixed in equal volumes using a tissue homogenizer and adequately emulsified, The prepared collagen emulsion was stored at 4 °C in a refrigerator for model establishment. For primary immunization (Day 0), the collagen emulsion (0.2 mL) was administered via intradermal injection at 1–2 cm from the root of the rat tail. For booster immunization (Day 7), 0.1 mL of the emulsion was intradermally injected at the same site (1–2 cm from the tail root) per rat. Rats in the control group were injected with normal saline in the same manner and volume.

### Anti-RA pharmacodynamics study

#### Animal grouping and treatment

Rats were randomly divided into 6 groups, Control, CIA group, MTX group (1.5 mg∙kg^−1^), HC-L group (0.0948 g∙kg^−1^), HC-M group (0.1896 g∙kg^−1^), and HC-H group (0.3792 g∙kg^−1^), with 12 rats in each group. In this experiment, the high-dose group was set at 1/5 of the Lethal Dose 50% (LD_50_) [12] of HC, the medium-dose group at 1/10 of LD_50_, and the low-dose group at 1/20 of LD_50_. On the 10th day after the initial immunization, rats in each group were administered the drug orally at a dose of 10 mL/kg. The HC group received the drug once daily, the MTX group once weekly, for a total of 5 weeks. The control group and model group were administered a 0.5% CMC-Na solution.

#### Measurement of toe swelling

Toe swelling was measured by vernier caliper every 7 days, and the average value of bilateral hindfoot measurement was used as the evaluation index of individual foot swelling.

#### Arthritis index assessment

A double-blind evaluation mechanism was employed, with independent observers quantifying joint lesions in CIA model rats using a standardized 5-point scoring system. The AI value was determined based on the cumulative score of all four limbs, with 0 ≤ AI ≤ 16 (4 points × 4 limbs), to assess the effect of the HC on joint lesions in CIA rats. When the AI value ≥ 4, the model was deemed successful [13]. The scoring table is shown in Table 1.

**Table 1:** AI scoring criteria

Score	AI standards
0	Normal
1	One or more toe joints are swollen or inflamed
2	Moderate redness and swelling below the ankle joint
3	Severe redness and swelling including the knee joint
4	Including the knee joint, there is complete redness and swelling, joint deformation, and disability

#### Measurement of immune organ index

After the last dose, rats were anesthetized with 1% sodium pentobarbital (40 mg/kg). After blood was collected from the abdominal aorta of rats in each group, the thymus and spleen were removed and weighed, and the thymus and spleen indexes were calculated to observe the effect of HC on the immune organs of CIA rats.

#### HE staining and cytokine detection

The slices were dried at 60 °C for 2 h. Then it was deparaffinized, stained with hematoxylin for 3–8 min, differentiated with 1% hydrochloric ethanol, stained with eosin for 3 min, dehydrated, sealed with neutral gum, and observed under a microscope. ELISA kits were used to determine the levels of MMP-3, MMP-2, TNF-α, and IL-6 in the synovium.

### Cardiac toxicity study

#### Animal grouping and treatment

The high-dose group (0.0294 g∙kg^−1^) was given 1/5 of the LD_50_ of RC for 5 weeks to establish the cardiotoxicity model of RC, The animals were divided into five groups, CIA model group, RC group, HC-H group, HC-M group, and HC-L group. The body weight of the rats was recorded from day 0 and every 7 days for 7 weeks. After the last administration, the rats were anesthetized by inhalation of isoflurane, fixed on the rat plate, and electrocardiogram (ECG) was recorded by a multi-channel physiological recorder. And in this study, initially 12 rats were included, but due to mortality, 10 rats were finally analysed in the ECG and toxicity study.

#### Serum biochemical testing

Blood was collected from anesthetized rats, centrifuged, and the supernatant was collected for testing of TBA, ALP, ALT, AST, CK, LDH and TP.

### Analysis by UPLC-Q-Orbitrap-MS/MS technology

#### Chromatographic and mass spectrometry conditions

Chromatographic conditions: ZOXExtend-C18 column (2.1 mm × 100 mm, 1.8 μm) mobile phase, 10 mmol ammonium acetate aqueous solution, ammonium acetate (A)-acetonitrile (B) gradient elution (0–0.3 min, 95% A; 0.3–0.5 min, 95–90% A; 0.5–18 min, 90–60% A; 18–19 min, 60–40% A; 19–20.5 min, 40–1% A; 20.5–24 min, 1% A; 24–24.3 min, 1%–95% A), column temperature, 40 °C, flow rate, 0.3 mL∙min^−1^, injection volume, 2 μL.

Mass spectrometry conditions: Electrospray ionization (ESI) was used, with positive and negative ion detection modes. Nitrogen was used as the desolvation gas, with a drying gas temperature of 450 °C and a drying gas flow rate of 8 L min^−1^. The capillary voltage was 0.1 kV, the cone voltage was 40 V, and the collision energy was 15–45 V. The scanning mode was full scan, with a mass scanning range of m/z 50–1200.

#### In vitro compositional analysis

HC were crushed, wetted with 1 g of 25% to 28% concentrated ammonia solution and sonicated with 25 mL of diethyl ether. The supernatant was evaporated to dryness under reduced pressure, redissolved with acetonitrile, filtered through a 0.22 μm microporous filter, and finally analyzed by liquid chromatography-mass spectrometry.

#### In vivo compositional analysis

The rats in the HC group (0.0948 g∙kg^−1^) were given 10 mL∙kg^−1^ by gavage once a day for 5 weeks. The control group and the model group were given 0.5% CMC-Na solution. Anesthesia was performed 1 h after the last administration, blood was collected from the abdominal aorta, and heart tissue was removed. 200 μL of drug-containing plasma and heart homogenate were placed in a centrifuge tube, fourfold acetonitrile was added, vortexed for 1 min, and centrifuged. The supernatant was transferred to a 1.5 mL centrifuge tube, blow-dried under nitrogen gas, redissolved with 100 μL of 70% acetonitrile, centrifuged, and the supernatant was taken for LC–MS analysis. The control group was treated as above.

#### Analysis of composition data in vitro and in vivo

According to the relevant literature, the chemical composition database of *Caowu* was established, which mainly included compound name, molecular formula, relative molecular mass and mass-to-charge ratio. Thermo Xcalibur software was used for data acquisition and analysis. Firstly, the extract function was used to find compounds. Secondly, according to the compounds found, the precision mass number was selected within the range of ± 10 ppm, and the target screening function was used for matching screening. Finally, compared with the database, combined with the fragmentation law, the structure of the components in vitro and in vivo was identified.

### Proteomic analysis

Take three samples from each group. Protein samples were initially extracted, and their concentrations were determined. Based on the quantification results, equal amounts of protein were subjected to enzymatic digestion. The resulting peptides were reconstituted in mobile phase A and separated using a NanoElute UHPLC system. The mobile phase A was an aqueous solution containing 0.1% formic acid and 2% acetonitrile. Mobile phase B consisted of acetonitrile–water soluble solution containing 0.1% formic acid. The liquid phase gradient was set to 0–14 min, 6%-24%B; 14–16 min, 24%-35%B; 16–18 min, 35%-80%B; The flow rate was maintained at 500 nL∙min^−1^ for 18 to 20 min at 80%B. After chromatographic separation, the samples were ionized by capillary electrospray ion source and imported into timsTOF Pro mass spectrometer for acquisition in dia-PASEF mode. The primary scan range was 300–1500 m/z, and the secondary fragment window was 400–850 m/z. Each MS1 spectrum was triggered for 20 PASEF acquisition, and the TOF detector simultaneously captured the parent ion and fragment ion information.

Group t-test was performed on the protein quantitative data of the two groups of samples to be compared. In data analysis, proteins with *P* ≤ 0.05 and a fold change of more than 1.2 (synovium), *P* ≤ 0.05 and a fold change of more than 2 (cardiac) between the two groups were selected as candidate differential proteins. Specifically, FC > 1.2 indicates significant upregulation for synovium, FC < 1/1.2 ≈ 0.833 indicated significant down-regulation. Similarly, for the heart, FC > 2 indicates significant up-regulation, and FC < 1/2 = 0.5 indicates significant down-regulation. The differentially expressed proteins were analyzed using Omics Box, for GO enrichment analysis, annotation was performed using eggnog-mapper (v2.1.6) software, KEGG pathway analysis was annotated using diamond (v2.0.11.149) software.

DIA data were searched using the DIA-NN (v 1.8) search engine with default software parameters. The database used was Rattus_norvegicus_10116_PR_20231121.fasta (47,943 sequences). The enzyme digestion method was set to Trypsin/P, and the maximum missed cleavage count was set to 1. Fixed modifications were set as: N-term M excision and C carbamidomethylation. A theoretical spectral library was constructed using deep learning algorithms, with reverse searching added to calculate the false discovery rate (FDR) caused by random matches. The FDR for precursor identification was set to 1%.

### Western blot

Take three samples from each group. Samples were first lysed for protein extraction. After centrifugation at 4 °C for 20 min, the protein concentration was determined and denatured by boiling. Denatured protein samples were separated by electrophoresis, transferred to membranes, and blocked. Diluted primary antibodies Nrf2 (1:5000, v:v), HO-1 (1:2000, v:v), SOD1 (1:5000, v:v), Beta actin (1:5000, v:v), Caspase 3 (1:2000, v:v), Bax (1:2000, V: V) and Bcl2 (1:5000, v:v) were added and incubated overnight. The corresponding secondary antibody (1:2000, v:v) was added and incubated for 1 h at room temperature. ECL chemiluminescence droplets were added to the membrane, and the bands were automatically exposed and developed by the ChemiDoc TMMP system. Image J software was used to quantify the results.

### Molecular docking validation

The blood components and heart components of HC were docked with the core therapeutic targets in sequence. The 3D structures of the active ingredients were downloaded from the PubChem platform, and the protein structures of the targets were obtained from the PDB database. Using Discovery Studio 2019 software, routine preprocessing of molecular structures was performed, all water molecules were removed, force field optimization was performed after hydrogenation, and the docking scores of the receptor and ligand were recorded.

### Statistical analysis

SPSS 19.0 software was used for statistical analysis, and all data were described by Mean ± standard deviation (Mean ± SD). One-way ANOVA was used for comparisons among multiple groups. When variances were homogeneous, the LSD test was applied, when variances were heterogeneous, the rank sum test was used. Repeated measures ANOVA was used for comparisons among different days, and *P* < 0.05 was considered statistically significant.

## Results

### Pharmacodynamic study of HC in CIA rats

**Fig. 1:**
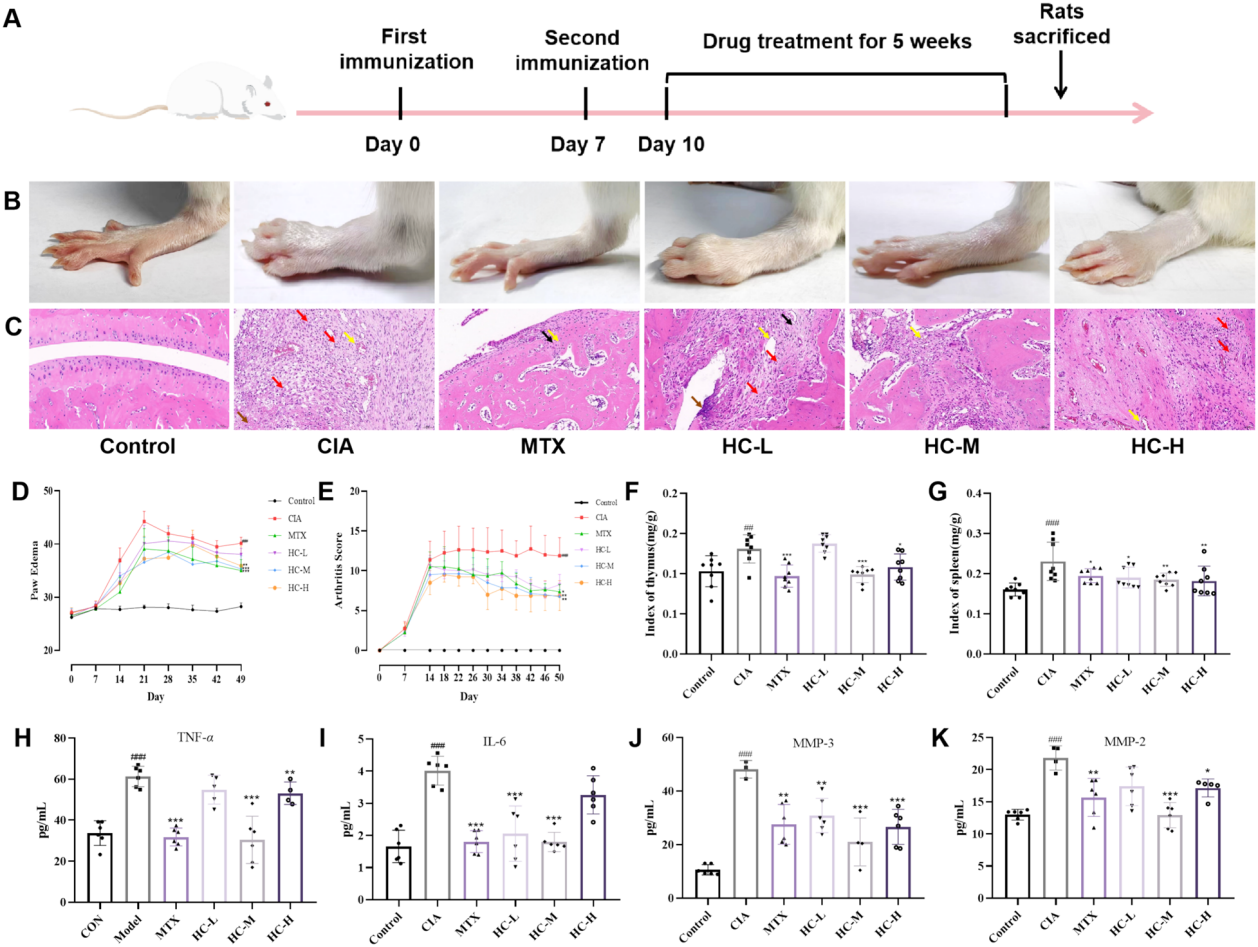
Pharmacodynamic Study of HC in CIA Rats **A** Schedule chart; **B** Representative photographs of hind paws at the end of each experiment; **C** Synovial tissue was stained with HE; **D** Bilateral foot swelling in different groups; **E** AI scores in different groups; **F** Indices of thymus in different groups; **G** Indices of spleen in different groups; **H**–**K** The expression of inflammatory factors TNF-α, IL-6, MMP-3 and MMP-2 in different groups. All data are presented as mean ± SD, compared with the control group, ^###^*P* < 0.001; compared with CIA group, ^*^P < 0.05, ^**^P < 0.01, ^***^P < 0.001

To verify the therapeutic efficacy of HC for arthritis, we administered different doses of HC. The timeline is shown in Fig. 1A. We observed that the control group rats showed no swelling in all four paws, compared with the control group, the model group rats exhibited swelling and ulceration of the hind paws accompanied by lameness, with the swelling reaching its peak on day 21 (*P* < 0.01), compared with the model group, by the fifth week of administration, rats in the HC-treated group, and the positive control group showed significant inhibition of swelling in the hind paws, with the moderate-dose group exhibiting the most pronounced inhibition (*P* < 0.01) (Fig. 1B, D), however, the efficacy of HC-H group actually declined and was even lower than that of HC-M group. Therefore, the efficacy of this drug does not follow the traditional linear dose-dependent pattern, instead, there exists an optimal effective dose range. HE staining of the ankle joint revealed that in the control group, the cartilage surface of the ankle joint was smooth and flat, with uniformly distributed chondrocytes, no synovial hyperplasia or inflammatory cell infiltration, and no secretions in the joint cavity. In contrast, the model group exhibited basic structural destruction of the joint, cartilage damage on the joint surface, extensive tissue necrosis, accompanied by vascular proliferation (yellow arrows), a small amount of exudate can be observed within the joint cavity (brown arrows) and inflammatory cell infiltration (red arrows). However, following HC intervention, the aforementioned pathological changes showed varying degrees of improvement. In the low-dose group, inflammatory cell infiltration remained visible, with a small amount of effusion present in the joint cavity and minimal connective tissue proliferation (black arrows). The medium-dose group demonstrated better efficacy, exhibiting only sparse neovascularization. In contrast, the high-dose group still showed inflammatory cell infiltration and significant neovascularization (Fig. 1C). Additionally, we observed a significant increase in the arthritis index in the model group rats. Compared with the model group, the arthritis index was reduced in the various drug groups (Fig. 1E). Meanwhile, we measured the thymus and spleen indices of the rats. Compared with the blank group, the thymus and spleen indices of the model group rats were significantly elevated (*P* < 0.01). Compared with the model group, the thymus and spleen indices of the rats in each treatment group were reduced (Fig. 1F, G). Regarding the detection of synovial cytokines, we can see that compared with the control group, the levels of MMP-2, MMP-3, TNF-α and IL-6 in the synovium of rats in the model group were significantly elevated (*P* < 0.001). Compared with the model group, the levels of MMP-2, MMP-3, TNF-α, and IL-6 were reduced in the HC-M group compared with the model group (*P* < 0.001) (Fig. 1H–K). These results indicate that HC alleviates paw swelling in CIA rats, reduces the arthritis index, and decreases the secretion of inflammatory factors MMP-2, MMP-3, TNF-α, and IL-6, suggesting that HC has anti-arthritic effects.

### Toxicodynamic study of HC in CIA rats

**Fig. 2:**
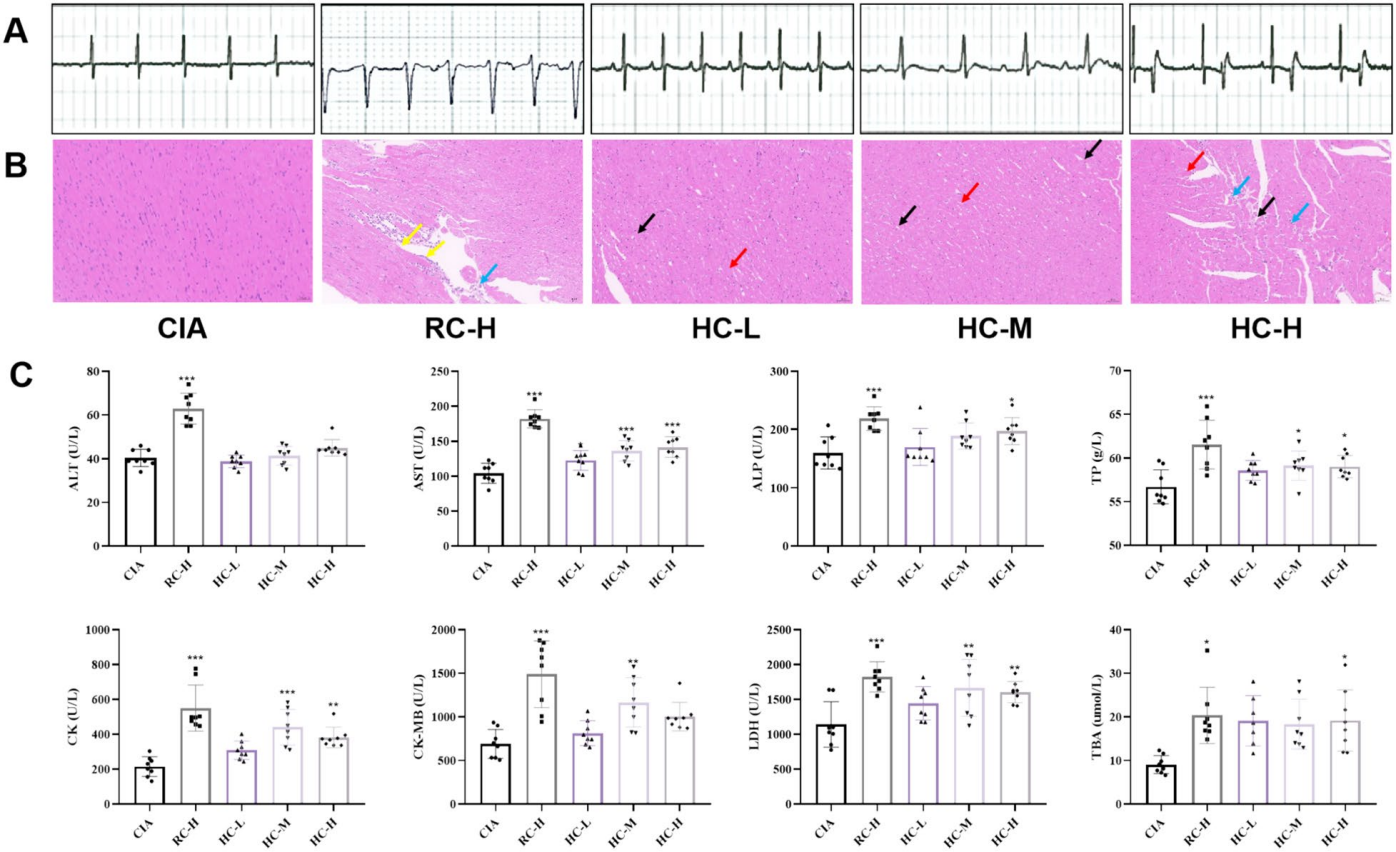
Toxicodynamic study of HC in CIA rats **A** Electrocardiogram of rats in different groups; **B** Cardiac tissues were stained with HE; **C** The expressions of ALT, AST, ALP, TP, CK, CK-MB, LDH and TBA in different groups were analyzed. All data are presented as mean ± SD, compared with CIA group, ^*^P < 0.05, ^**^P < 0.01, ^***^P < 0.001

Next, to investigate the toxicological effects of HC on the hearts of rats with CIA, we found that compared with the CIA group, the R-R interval, PR interval, QT interval, and QRS wave were prolonged in the ECG of rats in the RC group, and the heart rate was significantly reduced (*P* < 0.01). The ECG showed ventricular flutter, and the QRS wave was not visible. In the HC group, the R-R interval, PR interval, QT interval, and QRS complex were prolonged compared to the CIA group, with a significant decrease in heart rate (*P* < 0.05), as shown in Fig. 2A and Table 2. Pathological staining revealed that the endocardium, myocardium, and epicardium of the CIA group rats exhibited intact tissue structure integrity, with myofibrils arranged in a regular parallel pattern. Myocardial fibers demonstrated typical eosinophilic homogeneous staining characteristics. Collagen content in the interstitial tissue showed no pathological changes, and no focal necrosis or inflammatory cell infiltration was detected. In the high-dose RC group, local myocardial cell loss (yellow arrows), minimal connective tissue proliferation, widened myocardial interstices, irregularly arranged cells of varying sizes, and rare lymphocytes (blue arrows) were observed. In the low-dose and medium-dose HC groups, local loosely arranged, irregular myocardial cells (black arrows), widened myocardial interstices, and cells of varying sizes were observed, a small number of myocardial cells with striatal dissolution (red arrow), and enhanced eosinophilia. In the HC high-dose group, lymphocytes were rarely observed in the interstitium (Fig. 2B). Additionally, we measured relevant indicators and found that compared with the CIA group, the RC high-dose group showed significantly elevated levels of ALT, AST, ALP, TP, LDH, CK, CK-MB, and TBA in the blood (*P* < 0.05); In the HC treatment group, AST, ALP, TP, LDH, CK, CK-MB, and TBA levels were also generally elevated (*P* < 0.05), while ALT levels were not significantly different (Fig. 2C).

**Table 2:** The effect of HC on the electrocardiogram of CIA rats (n = 10)

	Heart Rate (BPM)	RR Interval (s)	PR Interval (s)	QRS Interval (s)	QT Interval (s)	JT Interval (s)
CIA	340.483 ± 14.724	0.160 ± 0.005	0.043 ± 0.002	0.013 ± 0.001	0.038 ± 0.002	0.018 ± 0.002
RC-H	304.240 ± 20.205^***^	0.202 ± 0.022^***^	0.052 ± 0.010^***^	0.026 ± 0.006^***^	0.051 ± 0.005^***^	0.025 ± 0.007^*^
HC-L	331.533 ± 12.461	0.165 ± 0.008	0.045 ± 0.002	0.018 ± 0.001^*^	0.044 ± 0.003^*^	0.024 ± 0.002
HC-M	328.000 ± 10.930	0.188 ± 0.012^***^	0.048 ± 0.001	0.022 ± 0.002^***^	0.050 ± 0.005^***^	0.026 ± 0.002^*^
HC-H	312.600 ± 8.106^**^	0.192 ± 0.005^***^	0.051 ± 0.003^**^	0.021 ± 0.002^***^	0.046 ± 0.003^**^	0.027 ± 0.004^**^

Compared with the CIA, *P < 0.05, **P < 0.01, ***P < 0.001

### Identification of in vivo and in vitro components

**Fig. 3:**
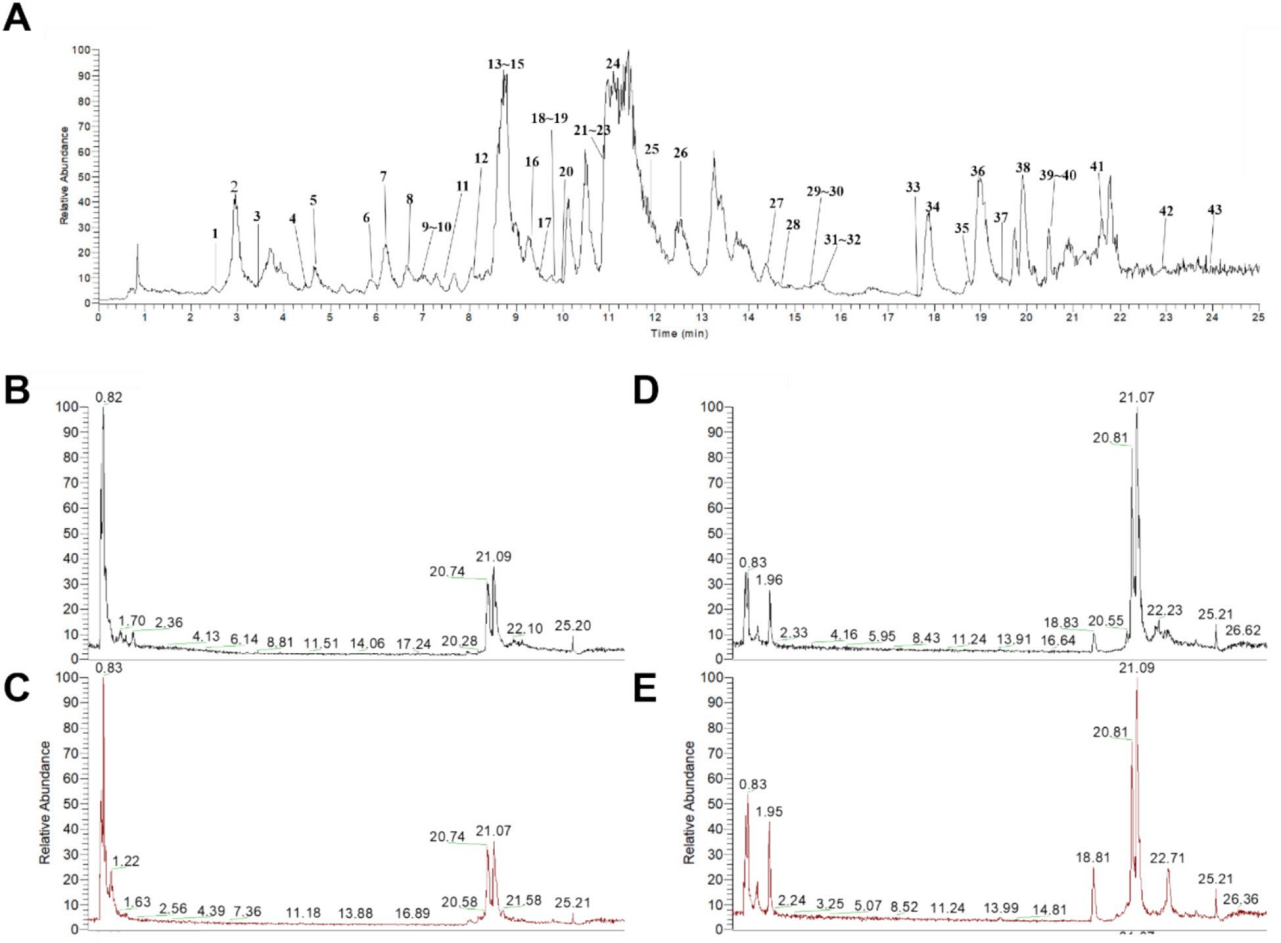
Identification of in vivo and in vitro components **A** TIC plot of HC; **B** TIC plot of blank plasma; **C** TIC plot of rat plasma after HC administration; **D** TIC plot of blank heart tissue; **E** TIC plot of rat heart after HC administration

This study employed UPLC-Q-Orbitrap MS/MS technology to analyze the components in vitro HC, in vivo plasma, and the primary toxic target organ—the heart. First, the total ion current chromatograms of HC in positive ion mode are shown in Fig. 3A. A total of 43 compounds were identified, with the identified compounds listed in Table 3.

**Table 3:** Results of the identification of HC chemical constituents

No	*t*_R_/min	Molecular formula	Adduct ion	*m*/*z* Measured value	*m*/*z* Theoretical value	Fragment ion	Chemical compound
1	2.49	C_24_H_39_NO_9_	[M + H]^+^	486.2684	486.2698	436.2316, 404.2056	Mesaconine
2	2.98	C_25_H_41_NO_9_	[M + H]^+^	500.2842	500.2854	450.2472, 468.2578	Aconine
3	3.54	C_22_H_35_NO_5_	[M + H]^+^	394.2578	394.2588	376.2472, 358.2367	Karakolidine
4	4.49	C_23_H_37_NO_6_	[M + H]^+^	424.2681	424.2694	406.2577	Senbusine A
5	4.67	C_22_H_35_NO_5_	[M + H]^+^	394.2579	394.2588	376.2474, 358.2370	Chuanfumine
6	5.59	C_22_H_33_NO_2_	[M + H]^+^	344.2574	344.2584	326.2468	Bullatine A
7	6.20	C_23_H_37_NO_6_	[M + H]^+^	424.2679	424.2694	406.2575	Senbusine B
8	6.64	C_25_H_41_NO_6_	[M + H]^+^	452.2995	452.3007	420.2733, 388.2471	Chasmanine
9	6.9	C_21_H_33_NO_4_	[M + H]^+^	364.2471	364.2482	346.2365	16-β-hydroxycardiopetaline
10	6.93	C_24_H_39_NO_5_	[M + H]^+^	422.2889	422.2901	390.2628	talatizamine
11	7.58	C_30_H_41_NO_9_	[M + H]^+^	560.2842	560.2854	528.2581	N-demethyl-14-benzoylhapoconine
12	8.25	C_24_H_35_NO_8_	[M + H]^+^	466.2428	466.2435	430.2578, 448.2680	14-dehydro-19-oxodelcosine
13	8.40	C_23_H_37_NO_5_	[M + H]^+^	408.2732	408.2744	390.2626	Isotalatizidine
14	8.72	C_22_H_33_NO_3_	[M + H]^+^	360.2519	360.2533	342.2416	Napelline
15	8.94	C_22_H_35_NO_4_	[M + H]^+^	378.2628	378.2639	360.2522	Karakoline
16	9.27	C_24_H_39_NO_7_	[M + H]^+^	454.2788	454.2799	436.2682/404.2424	Fuziline
18	9.45	C_31_H_43_NO_10_	[M + H]^+^	590.2946	590.2960	540.2578/558.2668	14-benzoylmesaconine
17	9.50	C_23_H_33_NO_6_	[M + H]^+^	420.2367	420.2381	402.2263	Giraldine F
44	9.60	C_31_H_43_NO_11_	[M + H]^+^	606.3683	606.2909	588.2787, 556.2794	14-benzoyl-10-OH-mesaconine
19	9.95	C_30_H_41_NO_10_	[M + H]^+^	576.2790	576.2803	558.2681, 494.2159	N-deethyl-14-benzoylaconine
21	10.38	C_31_H_43_NO_9_	[M + H]^+^	574.2998	574.3011	542.2736	14-benzoylhypaconine
20	10.48	C_24_H_39_NO_6_	[M + H]^+^	438.2837	438.2850	420.2732, 388.2470	Neoline
22	10.76	C_24_H_39_NO_8_	[M + H]^+^	470.2737	470.2748	438.2473	Hypaconine
24	11.10	C_32_H_45_NO_10_	[M + H]^+^	604.3101	604.3116	554.2735, 572.2839	14-benzoylaconine
23	11.46	C_22_H_31_NO_3_	[M + H]^+^	358.2365	358.2377	340.2262	Songorine
25	12.54	C_32_H_43_NO_9_	[M + H]^+^	586.2996	586.3011	568.2891, 554.2733	Dehydrated benzoylaconine
26	14.27	C_31_H_41_NO_9_	[M + H]^+^	572.2841	572.2854	554.2735, 522.2473	Dehydrated benzoylmesaconine
27	14.42	C_32_H_43_NO_11_	[M + H]^+^	618.2892	618.2909	558.2685	N-demethyl-mesaconitine
28	14.69	C_33_H_45_NO_11_	[M + H]^+^	632.3049	632.3065	572.2841, 540.2574	Mesaconitine
29	15.26	C_33_H_45_NO_9_	[M + H]^+^	600.3153	600.3167	540.2944, 508.2681	13-deoxyhypaconitine
30	15.41	C_32_H_43_NO_10_	[M + H]^+^	602.2579	602.2960	406.1636	N-demethyl-hypaconitine
31	15.55	C_33_H_45_NO_10_	[M + H]^+^	616.3103	616.3116	556.2893, 524.2632	hypaconitine
32	17.94	C_34_H_45_NO_10_	[M + H]^+^	628.3100	628.3116	568.2891	3-dehydroaconitine
33	18.19	C_33_H_45_NO_12_	[M + H]^+^	648.2997	648.3015	588.2787, 556.2534	10-OH-mesaconitine
34	18.98	C_34_H_47_NO_11_	[M + H]^+^	646.3204	646.3222	586.2995, 554.2734	Aconitine
35	19.21	C_34_H_47_NO_12_	[M + H]^+^	662.3152	662.3171	602.2943, 570.2682	Aconifine
36	19.96	C_34_H_47_NO_10_	[M + H]^+^	630.3256	630.3273	570.3047, 538.2786	3-deoxyaconitine
37	20.2	C_33_H_43_NO_10_	[M + H]^+^	614.3307	614.2960	554.3099	dehydro-mesaconitine
39	20.51	C_32_H_45_NO_8_	[M + H]^+^	572.3203	572.3218	540.2940, 508.2679	14-O-anisoylneoline
40	20.57	C_31_H_41_NO_8_	[M + H]^+^	556.2893	556.2905	524.2632	Dehydrated benzoylhypaconine
41	21.8	C_50_H_75_NO_11_	[M + H]^+^	866.5012	866.5413	586.2685, 526.2416	8-linoenic acid-benzoylaconine
42	22.9	C_47_H_73_NO_11_	[M + H]^+^	828.6017	828.5256	572.3099, 309.2777	8-palmitic acid-benzoylmesaconine
43	23.96	C_49_H_71_NO_11_	[M + H]^+^	850.5437	850.5100	570.3045	8-linoleic acid-benzoylmesaconine

In positive ion mode, the total ion current chromatograms of HC in rat plasma at 1 h are shown in Figs. 3B–C. After blank plasma matrix background subtraction, a total of 24 parent compounds were identified in the drug-containing plasma. The alkaloid components in the plasma were classified, including 3 diester-type alkaloids, 4 monoester-type alkaloids, and 14 alcohol amine-type alkaloids. The total ion current chromatogram of HC in rat hearts after 1 h is shown in Fig. 3D–E. After blanking out the background of the blank heart matrix, a total of 25 parent compounds were identified in the drug-containing hearts. The alkaloid components in the drug-containing hearts were classified, with HC containing 3 diester-type alkaloids, 4 monoester-type alkaloids, and 15 alcohol amine-type alkaloids. The identified compounds are listed in Table 4.

**Table 4:** Results of the identification of the plasma and cardiac chemical composition of HC

No	Name	MS1	MS2	plasma	cardiac	Type
1	Ellagic acid	300.9983	245.0014	+	+	d
2	Songorine	358.2379	340.3291	+	+	c
3	Senbusine A	424.2699	406.2326	+	+	c
4	Neoline	438.2847	420.3199	+	+	c
5	N-demethyl-mesaconitine	618.2903	558.2254	+	−	a
6	N-demethyl-14-benzoylhapoconine	560.2876	528.3833	+	−	b
7	N-deethyl-14-benzoylaconine	576.2785	558.5848	+	+	b
8	Napelline	360.2536	342.3372	+	+	c
9	Karakoline	378.2641	360.2547	+	+	c
10	Karakolidine	394.2591	376.2227	+	+	c
11	Isotalatizidine	408.2738	390.3074	+	+	c
12	Isochebulic acid	355.0299	337.0083	+	+	d
13	Hypaconitine	616.3103	524.3708	+	+	a
14	Hypaconine	470.2758	438.3021	-	+	c
15	Glucose gallate tannin	331.0668	205.0342	+	+	e
16	Giraldine F	420.2377	402.3564	+	+	c
17	Dehydrated benzoylhypaconine	556.2934	524.3718	−	+	b
18	Dehydrated benzoylaconine	586.3000	536.1410	+	+	b
19	Chasmanine	452.3009	420.3199	+	+	c
20	Bullatine A	344.2585	326.2326	+	+	c
21	Aconitine	646.3228	554.0873	+	+	a
22	Aconine	500.2858	450.1911	+	+	c
23	3-deoxyaconitine	630.3282	538.3707	−	+	a
24	16-β-hydroxycardiopetaline	364.2479	346.0321	+	+	c
25	15-hydroxyneoline /fuziline	454.2796	436.3429	+	+	c
26	14-dehydro-19-oxodelcosine	466.2416	430.0886	+	+	c
27	14-benzoylaconine	604.3123	554.9702	+	+	b

a. Diester alkaloids; b. Monoester alkaloids; c. Alkyamine alkaloids; d. Phenolic acid; e. polyphenol

### Proteomics study of the efficacy of HC on CIA rat models

**Fig. 4:**
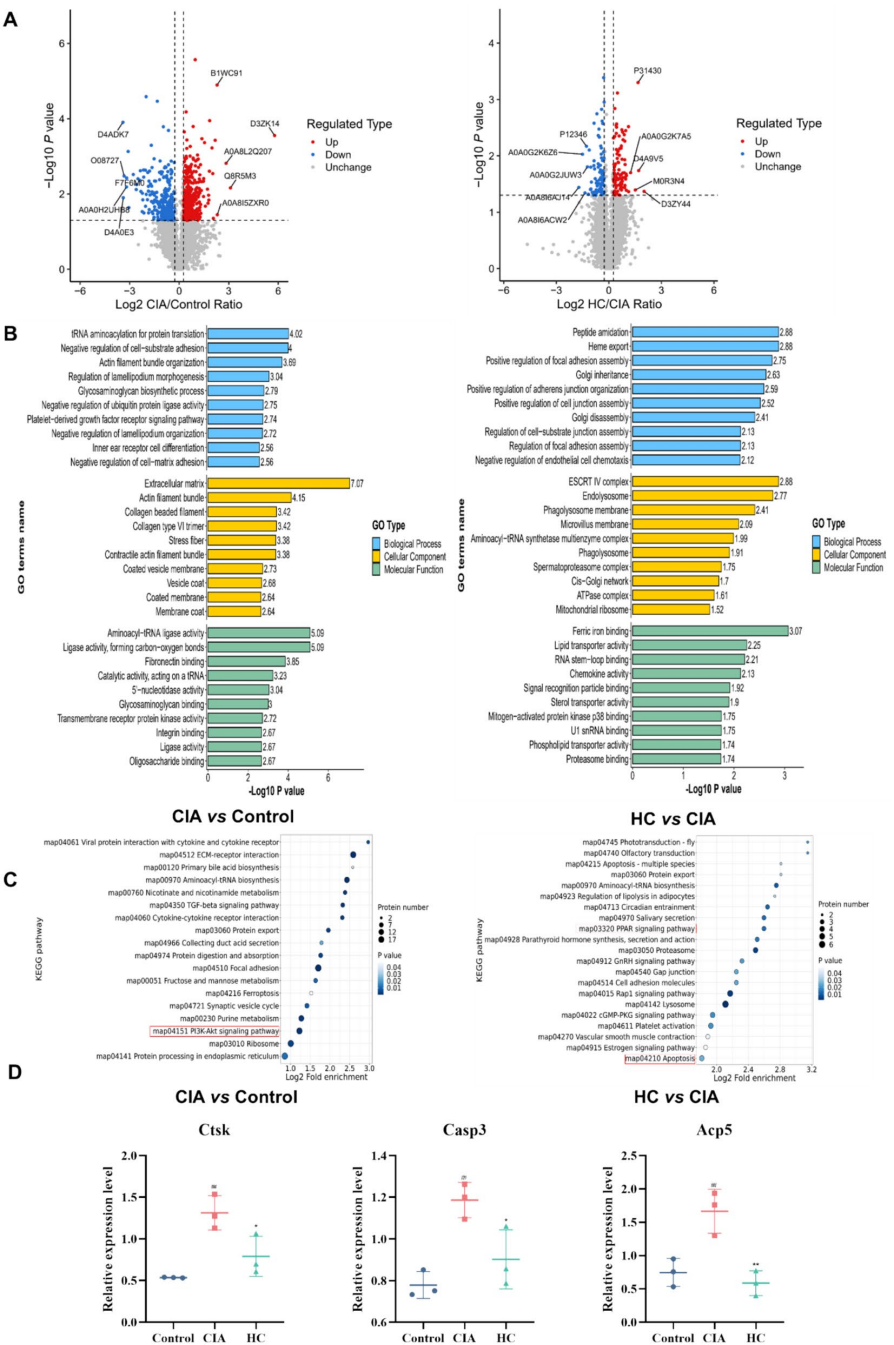
Proteomics study of HC on CIA rat model **A** Volcano map results; **B** GO enrichment analysis between different groups; **C** KEGG enrichment analysis of differentially expressed proteins in each group; **D** Specific protein expression of Ctsk, Casp3, and Acp5. All data are presented as mean ± SD (n = 3), compared with the control group, ^##^*P* < 0.01; compared with CIA group, ^*^P < 0.05, ^**^P < 0.01

To further assess the mechanism of action of HC on rheumatoid arthritis from a comprehensive perspective, we employed high-throughput proteomics technology to conduct a comprehensive analysis of the proteome of synovial tissue from CIA rats following HC administration, the specific proteome list is presented in Table S1. as shown in Fig. 4A. The volcano plot results revealed that, compared to the Control group, the CIA group exhibited 429 upregulated proteins and 256 downregulated proteins. In the CIA group compared to the HC group, 97 proteins were upregulated and 86 were downregulated. To further analyze the functional roles of the differentially expressed proteins, we performed functional enrichment analysis of the differentially expressed proteins in the CIA-Control and HC-CIA groups using the Blast2GO functional annotation module, as shown in Fig. 4B. Subsequently, KEGG enrichment analysis of the differentially expressed proteins in each group identified relevant pathways, with the CIA-Control group showing enrichment in pathways such as the PI3K-Akt signaling pathway. The enriched pathways for the HC-CIA group included the PPAR signaling pathway and the apoptosis pathway, among others (Fig. 4C). Next, we performed an intersection comparison of the differentially expressed proteins across groups. Finally, three significant proteins Ctsk, Casp3 and Acp5 were preliminarily screened out. (Fig. 4D).

### Proteomics study of the cardiotoxicity of HC in CIA rat models

**Fig. 5:**
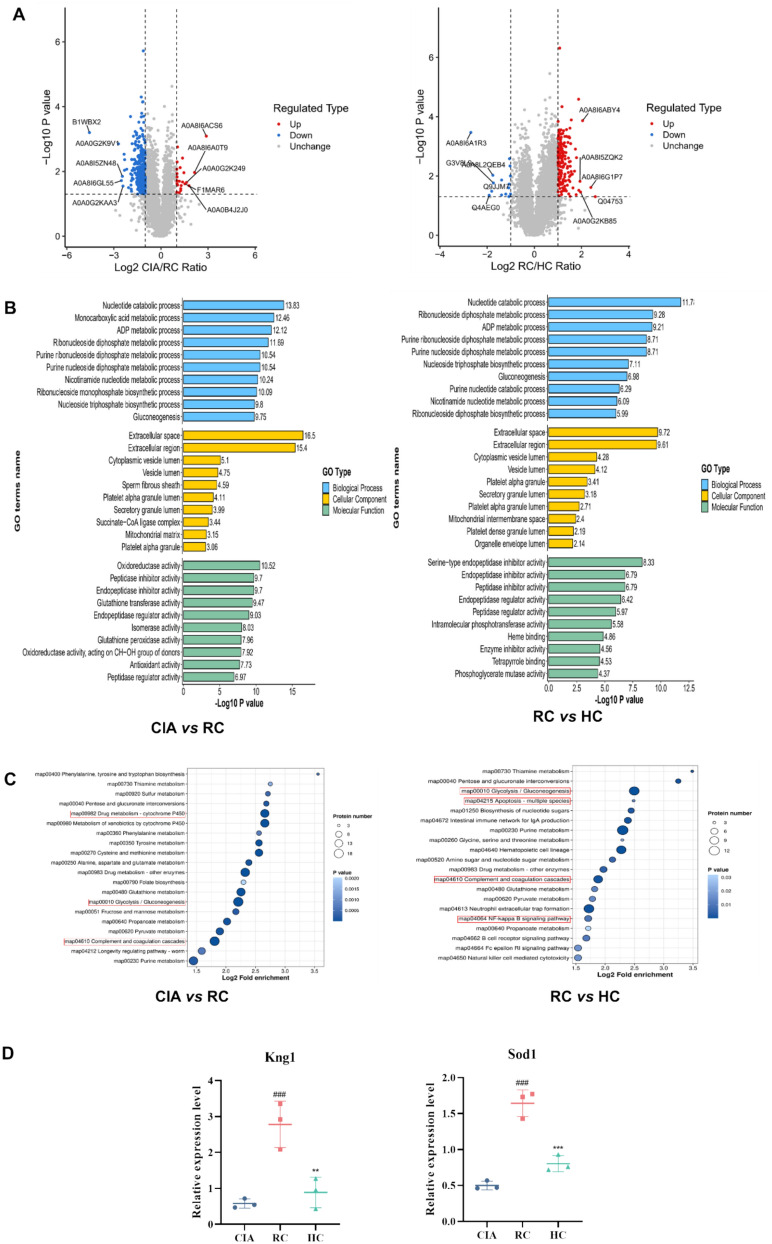
Proteomic study of HC cardiotoxicity in CIA rats **A** Volcano map results; **B** GO enrichment analysis between different groups; **C** KEGG enrichment analysis of differentially expressed proteins in each group; **D** Specific protein expression of Kng1 and Sod1. All data are presented as mean ± SD (n = 3), compared with the control group, ^###^*P* < 0.001; compared with SC group, ^**^P < 0.01 and ^***^P < 0.001

To investigate the cardiac toxicity of HC in CIA rats, we also conducted proteomics research to explore its mechanism of action. The specific list of proteins can be found in Table S2. As shown in Fig. 5A, there were 23 upregulated proteins and 385 downregulated proteins in the SC group vs. the CIA group; there were 180 upregulated proteins and 15 downregulated proteins in the HC group vs the SC group. To further analyze the functional roles of the differentially expressed proteins, we performed functional enrichment analysis on the differentially expressed proteins in the CIA-RC and RC-HC groups using the Blast2GO functional annotation module, as shown in Fig. 5B. Subsequently, KEGG pathway enrichment analysis of the differentially expressed proteins in each group revealed that the pathways in the SC-CIA group included Drug metabolism—cytochrome P450, Glycolysis/Gluconeogenesis, and Complement and coagulation cascades, among others. The pathways associated with the differentially expressed proteins in the HC-SC group included Glycolysis/Gluconeogenesis, apoptosis—multiple species, complement and coagulation cascades, and NF-kappa B signaling pathway, among others (Figs. 5C and D). After performing an intersection comparison of the differentially expressed proteins across all groups, two significant proteins, Kng1 and Sod1 were preliminarily identified.

### WB verification results

**Fig. 6:**
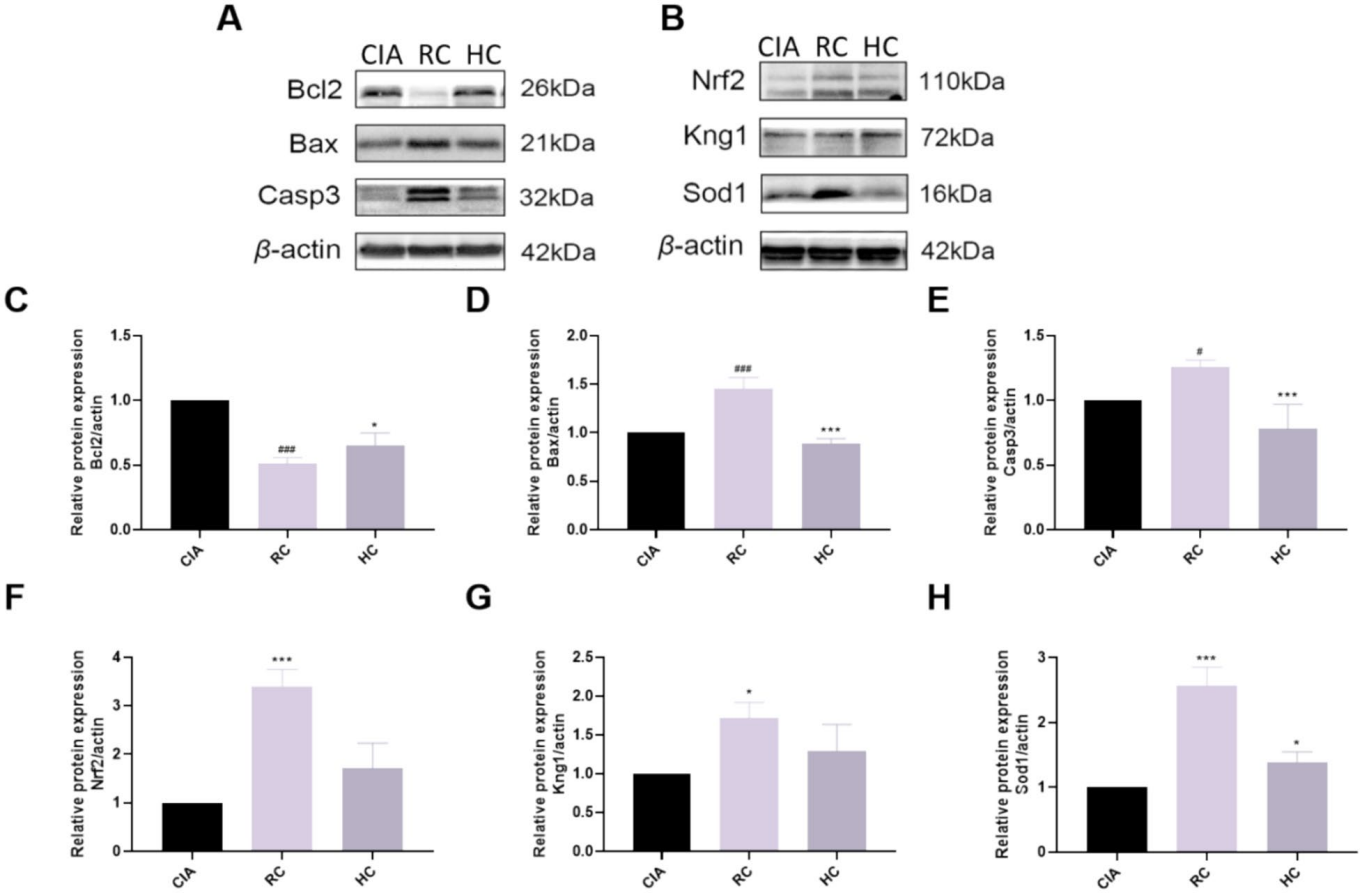
Western blot was used to detect related proteins **A** The expression of apoptotic factors Casp3, Bax and Bcl2 in cardiac tissue of different groups; **B** The expression of antioxidant factors Nrf2, kng1 and Sod1 in heart tissues of different groups were detected; **C**–**H** Results of quantitative analysis of Casp3, Bax, Bcl2, Nrf2, kng1 and Sod1. All data are presented as mean ± SD (n = 3), compared with the control group, ^#^*P* < 0.05 and ^###^*P* < 0.001; compared with CIA group, ^*^P < 0.05 and ^***^P < 0.001

To validate the effect of HC on cardiac toxicity, bioinformatics analysis of differentially expressed proteins revealed that the NF-*κ*B signaling pathway, apoptosis, and complement and coagulation cascades were highly enriched, suggesting that they may play a crucial role in HC's mitigation of cardiac toxicity. First, we detected apoptosis-related proteins and found that compared with the CIA group, the apoptosis factors Casp3 and Bax were significantly increased in the RC group (*P* < 0.05), while the anti-apoptotic factor Bcl2 was significantly decreased (*P* < 0.001). Compared with the RC group, the HC group showed significantly reduced levels of the apoptotic factors Casp3 and Bax (*P* < 0.001) and significantly increased levels of the anti-apoptotic factor Bcl2 (*P* < 0.001) (Fig. 6A, C–E). In addition, we found that compared with the CIA group, the factors Nrf2, Kng1 and Sod1 in the RC group were significantly increased (P < 0.05). Compared with the RC group, the expression of Nrf2, Kng1, and SOD1 was decreased in the HC group (Fig. 6B, F–H).

### Molecular docking verification results

**Fig. 7:**
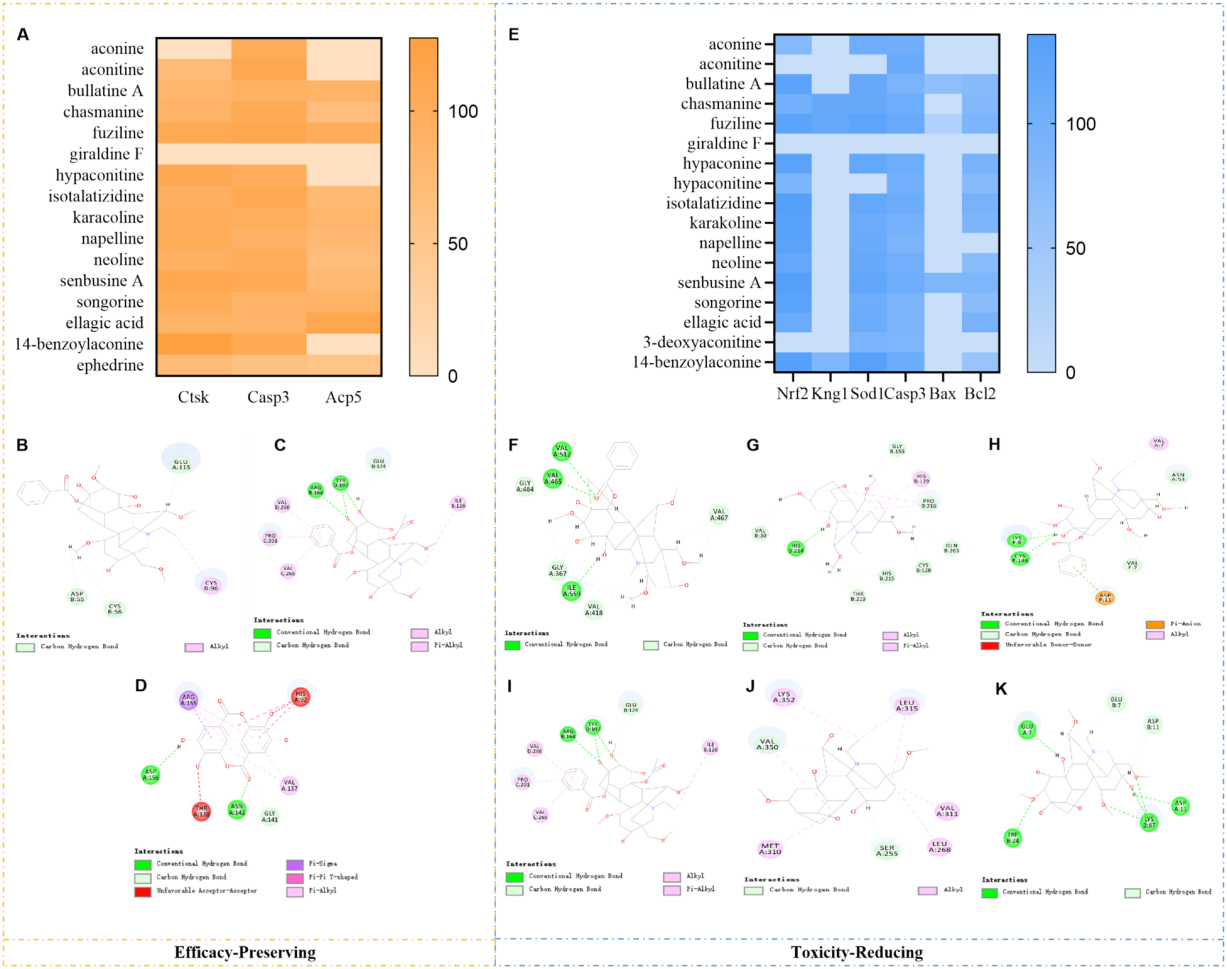
Analysis of molecular docking results (**A**) and (**E**) Docking scores of different proteins with active ingredients. **B** The 2D interaction diagrams of benzoylaconitine with the Ctsk protein. (**C**) and (**I**) The 2D interaction diagrams of aconitine with the Casp3 protein. **D** The 2D interaction diagrams of ellagic acid with the Acp5 protein. **F** The 2D interaction diagrams of benzoylmesaconine with the Nrf2 protein. **G** The 2D interaction diagrams of chasmanine with the Kng1 protein. **H** The 2D interaction diagrams of benzoylaconitine with the Sod1 protein. **J** The 2D interaction diagrams of Senbusine A with the Bax protein. **K** The 2D interaction diagrams of mesaconine with the Bcl2 protein

In order to further verify our conjecture, in the pharmacodynamic study of HC, we selected three key proteins Ctsk, Casp3 and Acp5 for molecular docking with the blood entry components of HC. First, we examined the docking scores of core targets and blood entry components, we chose ephedrine as the negative control. It can be concluded that Ctsk and benzoylaconitine, Casp3 and aconitine, Acp5 and ellagic acid bind well (Fig. 7A), and the binding mode is shown in Fig. 7B–D. Among them, benzoylaconitine interacted with Ctsk through ASP55, CYS56, GLU115 and CYS96. Aconitine binds to Casp3 through six residues including TYR197 and ARG164. Ellagic acid binds to Acp5 through seven residues including ARG155 and ASP156. Molecular docking of our selected six key proteins Nrf2, Kng1, Sod1, Casp3, Bax and Bcl2 with cardiac components was performed for the toxicology study in HC. It can be concluded that the docking scores of Nrf2 and benzoylmesaconine, Kng1 and chasmanine are high (Fig. 7E), and the binding conformation is shown in Fig. 7F-K. Specifically, benzoylmesaconine interacted with Nrf2 through seven residues, including VAL 512 and VAL 465. chasmanine binds to Kng1 through nine residues including CYS 128 and HIS 214. benzoylaconitine binds to Sod1 through six residues, including LYS 9 and CYS 146. Aconitine binds to Casp3 through six residues including TYR 197 and ARG 164. Senbusine A binds to Bax through seven residues including MET 310 and LYS 352. mesaconine binds to Bcl2 through four residues, GLU 7, TRP 24, LYS 87, and ASP 11. For the detailed 3D molecular docking diagrams, please refer to the supplementary materials (Figure S1).

## Discussion

**Fig. 8:**
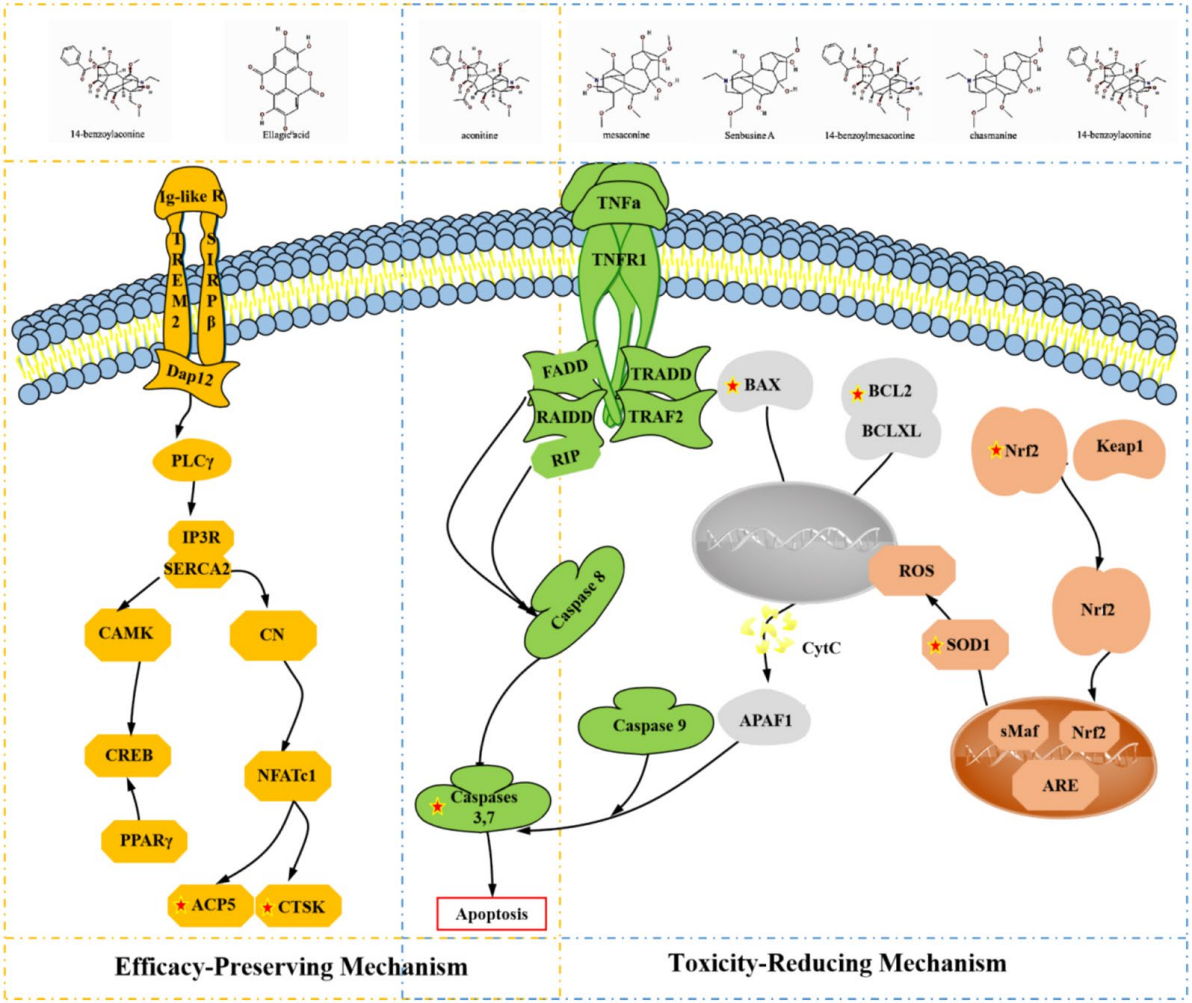
Diagram of mechanism

In recent years, TCM has gained popularity due to its good efficacy and few adverse reactions. However, the toxicity problem of TCM cannot be ignored. In order to further study the nature of its toxicity and its relationship with efficacy. Based on the anti-inflammatory activity of HC, this study constructed a dynamic "component-drug effect/toxic effect" network, integrated the analysis of blood components and heart components, and disease model-driven synovial and heart proteomics analysis to reveal the dynamic regulation mechanism of "anti-RA drug target activation-cardiotoxic pathway inhibition" (Fig. 8), and clarified the attenuation and efficacy principle of HC. It provides a new strategy for the study of attenuation and efficacy of toxic TCM processing.

In the pharmacodynamic evaluation of HC's efficacy in improving CIA rats, HE staining is a commonly used method in sectioning techniques and forms the basis of anatomical pathology diagnosis. In this experiment, we observed fundamental structural destruction in the joints of the model group. However, after treatment in each dosing group, symptoms were inhibited to varying degrees. Additionally, HC alleviated paw swelling and reduced the arthritis index in CIA rats. We found that the efficacy of this drug does not follow the traditional linear dose-dependent pattern. Instead, there is an optimal effective dose range, which is manifested as the medium dose in this research model. This might be due to the fact that at higher doses, the drug begins to exhibit potential toxicity or side effects, thereby affecting the overall physiological state of the animals and counteracting some of the therapeutic benefits. The low dose is safe, but the concentration of the active ingredient is insufficient to fully activate the main anti-inflammatory and related pathways, or it may only act on some targets, resulting in limited efficacy. The high dose, while significantly activating the anti-inflammatory pathways, begins to show the interference effect of toxicity, disrupting the overall synergistic network, leading to a decrease in efficacy or even an increase in toxicity [14].

It is reported that under pathological conditions, pro-inflammatory cytokines produced by synovial tissue, such as IL-6 and TNF-α, are positively correlated with the degree of cartilage damage, thereby affecting bone formation [15, 16]. Increasing evidence also indicates that highly expressed MMPs are key mediators responsible for the destruction of cartilage and non-osseous supporting structures in RA [17, 18]. Therefore, we examined relevant indicators and found that HC can reduce the secretion of inflammatory factors MMP-2, MMP-3, TNF-α, and IL-6. Among these, we observed that HC exhibited a more significant inhibitory effect on MMP-2 and MMP-3 at the same dosage, which may be attributed to its unique chemical composition profile. Previous studies have shown that HC is rich in ellagic acid, a compound that has been demonstrated to directly inhibit MMP activity [19]. These findings suggest that HC possesses anti-arthritic effects. Additionally, since HC is processed via immersion with Hezi juice, it retains some of the anti-inflammatory and analgesic activities of diester-type alkaloids under relatively mild conditions, while also introducing Hezi polyphenols to enhance antioxidant and matrix-protective functions [20]. This “toxicity-efficacy component balance” in the preparation process may also confer HC with greater advantages in alleviating inflammatory pain symptoms.

We then investigated the mechanism by which HC reduces cardiac toxicity. Based on our group's prior acute toxicity experiments, the LD₅₀ of RC was 0.147 g/kg, whereas that of HC was 1.896 g/kg, indicating that processing significantly broadens the therapeutic safety margin. In animal studies, experimental doses typically range from 1/10 to 1/20 of the safety threshold [21]. This study selected 1/5 of the LD₅₀ of RC as the positive control dose for cardiotoxicity to evaluate HC's dose-dependent protective effects. In this experiment, the RC group exhibited severe arrhythmias including ventricular flutter and QRS wave disappearance, along with significantly prolonged QT intervals, demonstrating marked cardiotoxicity [22, 23]. In the HC group, ECG parameter prolongation showed positive correlation with dosage, indicating residual diester alkaloids retain potential proarrhythmic risks, though with a significantly higher toxicity threshold than RC. Morphological analysis revealed well-preserved structural integrity in the endocardium, myocardium, and epicardium of the CIA group. The high-dose RC group exhibited myocardial cell loss, minor connective tissue proliferation, and irregular myocardial cell arrangement. The HC group showed localized irregular myocardial cell arrangement and widened myocardial interstices, indicating toxicity in both groups, though the RC demonstrated greater toxicity. Subsequent blood biochemical analysis revealed abnormalities in ALT, AST, CK-MB, and other indicators in the RC group, whereas HC group parameters improved, reflecting reduced myocardial cell damage after processing. This study demonstrates that RC is clearly unsuitable for long-term clinical administration due to severe cardiac toxicity and systemic side effects, whereas HC achieves a better balance between anti-inflammatory activity and cardiac toxicity.

This study employed 4D-FastDIA quantitative proteomics to investigate differential proteins in the synovium of CIA model rats treated with HC. Bioinformatics analysis of differentially expressed proteins revealed higher enrichment of the Phosphoinositide 3-kinase (PI3K)-Protein Kinase B (Akt) signaling pathway in the model group compared to the control group. The PI3K-Akt signaling pathway has been reported to play an indispensable role in rheumatoid arthritis. PI3K generates the second messenger PIP3 by phosphorylating phosphatidylinositol, thereby activating downstream Akt. Akt exerts biological effects by phosphorylating downstream targets, regulating cell survival, proliferation, metabolism, and inflammatory responses [24, 25]. Compared to the model group, the PPAR signaling pathway showed higher enrichment in the HC group. The PPAR pathway exhibits therapeutic potential by inhibiting inflammation and immune dysregulation through multiple mechanisms. PPAR belongs to the nuclear receptor family, comprising three subtypes: PPARα, PPARγ, and PPARδ. These receptors regulate target gene expression by binding ligands, participating in metabolism, inflammation, and immune modulation [26]. Among these, PPARγ reduces the production of pro-inflammatory cytokines such as TNF-α, IL-6, and IL-1β by antagonizing transcription factors like NF-κB [27]. Intersection analysis of differentially expressed proteins reversed in the treatment group preliminarily identified three significant proteins, Cathepsin K (Ctsk), Caspase-3 (Casp3), and (Acid phosphatase 5) Acp5. Ctsk, secreted by osteoclasts, degrades type I collagen in the bone matrix and directly contributes to bone resorption [28]. Casp3, a core enzyme in the apoptosis signaling pathway, plays a crucial role in arthritis development through apoptosis induced by its activation [29, 30], Acp5, highly expressed in osteoclasts, participates in bone matrix demineralization and degradation. Clinically, it serves as a biomarker for bone destruction, monitoring disease progression or treatment response [31, 32]. In this study, we found that HC administration significantly reduced Ctsk and Acp5 protein expression, inhibiting bone erosion; simultaneously, it markedly decreased Casp3 protein expression, suppressing apoptosis. Molecular docking was performed between the bioactive components of HC and three key proteins, Ctsk, Casp3, and Acp5. Among these, Ctsk exhibited the highest docking score with benzoylaconitine, suggesting it may inhibit collagen hydrolysis by occupying the active site of Ctsk, thereby slowing joint destruction. The high docking score between aconitine and Casp3 may indicate that aconitine activates the pro-apoptotic function of Casp3, inducing apoptosis in hyperproliferative synovial cells and thereby inhibiting joint cavity invasion. The high binding energy between ellagic acid and Acp5 may indicate that ellagic acid inhibits the active site of Acp5, blocking osteoclast maturation and reducing bone loss. In addition, the above three active ingredients benzoylaconitine, aconitine and ellagic acid were also reported to improve the inflammatory condition of RA, reduce the expression of inflammatory factors such as TNF-α and IL-1β, and have a positive effect on alleviating bone damage [33–35].

This study also conducted proteomics analysis on the mechanism by which HC alleviates cardiac toxicity. Compared with the RC group, the HC group showed significant enrichment in the NF-κB signaling pathway, apoptosis, and complement and coagulation cascade pathways. The NF-κB pathway promotes inflammatory cytokine release and contributes to cardiac injury; ROS accumulation can also activate this pathway, exacerbating inflammatory responses [36]. Apoptosis activates Caspase-3/7 via the mitochondrial or death receptor pathways, leading to cardiomyocyte death [37–39]. The complement and coagulation systems aggravate cardiac injury by promoting inflammation and thrombosis [40]. Further screening identified two key proteins, Kng1 and Sod1. Kng1 exerts pro-inflammatory, pro-apoptotic, and pro-oxidative effects, with its overexpression exacerbating cardiac toxicity [41]. Sod1 and Nrf2 belong to the antioxidant system, mitigating oxidative stress [36, 42]. Western blot results showed that Nrf2, Kng1, and Sod1 were highly expressed in the RC group, suggesting that RC induced oxidative stress and activated antioxidant responses. In contrast, expression of these proteins decreased in the HC group, indicating that the processing reduced toxic alkaloid content, thereby alleviating oxidative stress. Simultaneous detection of apoptosis-related proteins revealed that compared to the CIA group, pro-apoptotic proteins Casp3 and Bax were significantly elevated in the RC group, while anti-apoptotic protein Bcl2 was significantly reduced. Conversely, the HC group exhibited decreased Casp3 and Bax levels alongside increased Bcl2 expression. It is speculated that RC activates the apoptosis pathway, while HC inhibits apoptosis. Molecular docking results indicate that benzoylmesaconine and benzoylaconitine may activate the Nrf2-Sod1 axis to enhance antioxidant capacity, chasmanine may regulate the kinin system, Aconitine directly binds to Casp3 to promote apoptosis, while senbusine A and mesaconine may exert anti-apoptotic effects by stabilizing Bcl2 and inhibiting Bax. It has been reported that benzoylmesaconine, one of the active components mentioned above, can alleviate the inflammatory progression of rheumatoid arthritis by targeting the pyroptosis pathway [43]. In contrast, there are currently fewer reports on the effects of mesaconine, chasmanine, and senbusine A in improving arthritis in CIA rats.

Therefore, in this study, we investigated the underlying mechanisms through in vivo pharmacodynamic and in vivo toxicology studies, laying the foundation for research on the “effect-toxicity balance” of other toxic drugs. It is worth noting that the global proteomics strategy employed in this study provided a comprehensive perspective on the attenuation mechanism of HC, but failed to elucidate the distribution changes of proteins at the subcellular level. Recent studies have emphasized that sequential separation or subcellular separation of cardiac tissues rich in myosin proteins and extracellular matrix content can provide a deeper understanding of their molecular mechanisms [44, 45]. Therefore, future research will benefit from the adoption of these advanced fractionation techniques to more precisely locate and functionally validate the candidate targets discovered in this study.

## Conclusion

In this study, based on the traditional Mongolian medical theory that HC moderates drug properties, reduces toxicity, and preserves efficacy, we systematically elucidated a new paradigm of the dynamic balance between cardiotoxicity and anti-RA efficacy, and innovatively proposed a synergistic strategy of “inhibiting toxicity pathways while retaining efficacy-related targets”. First, the in vivo distribution of chemical constituents of HC was investigated. Next, a rat model of CIA was established to evaluate the efficacy and toxicity of long-term HC administration. Finally, high-throughput proteomic analysis of synovial and cardiac tissues was conducted to elucidate the mechanism by which HC moderates drug properties, reduces toxicity, and preserves efficacy, which was further validated using molecular docking and western blotting. This study not only provides a scientific basis for optimizing the processing of *Caowu*, but also offers valuable references for research on the “efficacy–toxicity balance” of other toxic medicinal agents.

## Supplementary Information

Supplementary file 1.

Supplementary file 2.

Supplementary file 3.

## Data Availability

The core data of this work are provided in the manuscript. Further information will be provided upon reasonable request.

## Abbreviations

Acp5: Acid phosphatase 5
Casp3: Caspase-3
CIA: Collagen- induced arthritis
Ctsk: Cathepsin K
ECG: Electrocardiogram
HC: Hezi processed *Caowu*
IL-6: Interleukin-6
Kng1: Kininogen-1
Nrf2: Nuclear factor erythroid 2-related factor 2
RA: Rheumatoid arthritis
Sod1: Superoxide dismutase 1
TNF-α: Tumor necrosis factor-α
