# Supplementary material for: A proteomics-based study on the mechanisms of Terminalia chebula Retz processed Aconitum kusnezoffii Reichb against rheumatoid arthritis and its cardiotoxicity reduction
Source: Chin Med. 2026 Jan 9;21:21. doi: 10.1186/s13020-025-01306-8 (PMC12784562; doi:10.1186/s13020-025-01306-8)

Figure S1

1.The 2D and 3D interaction diagrams of benzoylmesaconine with the Nrf2 protein.


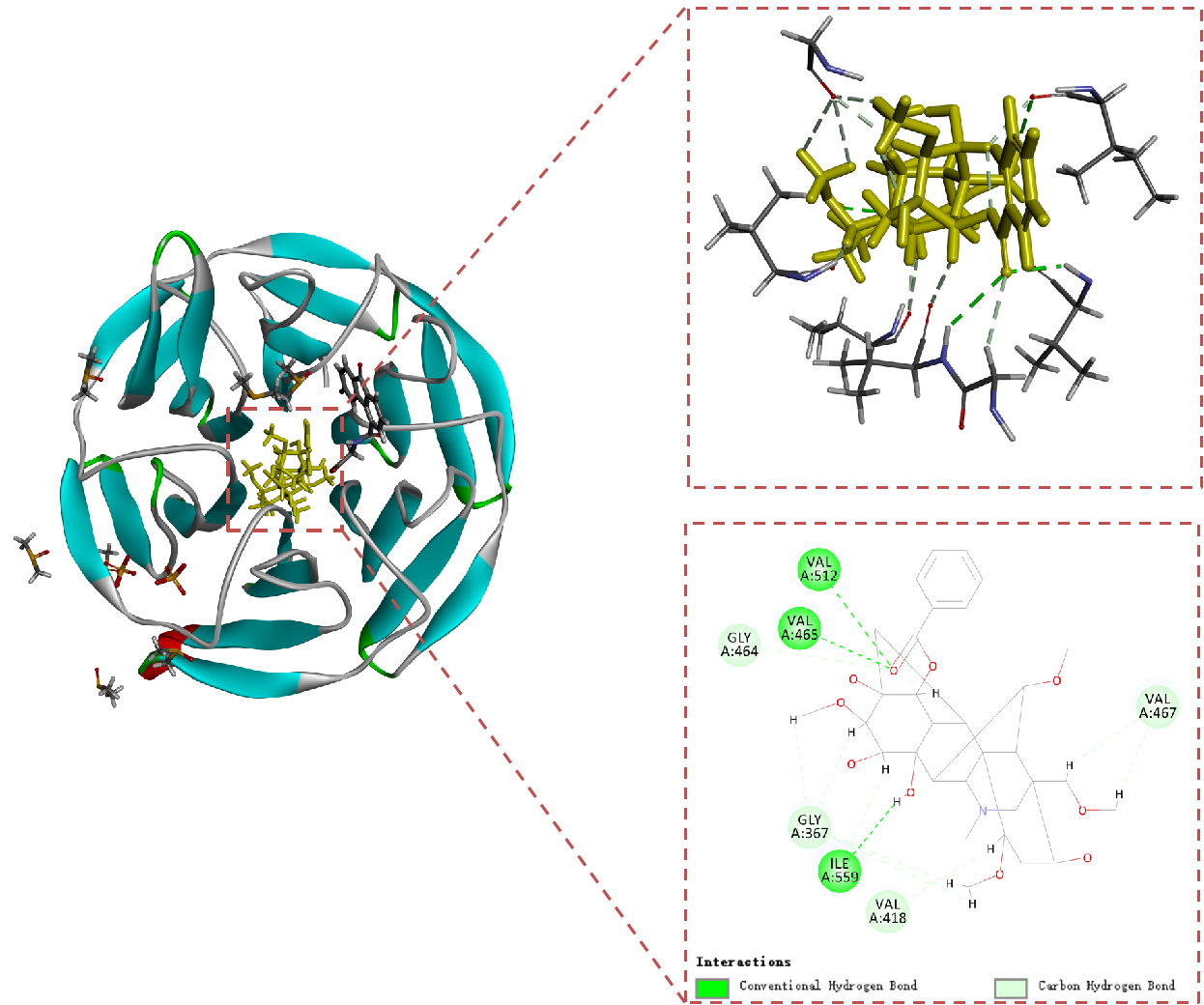


2.The 2D and 3D interaction diagrams of chasmanine with the Kng1 protein.


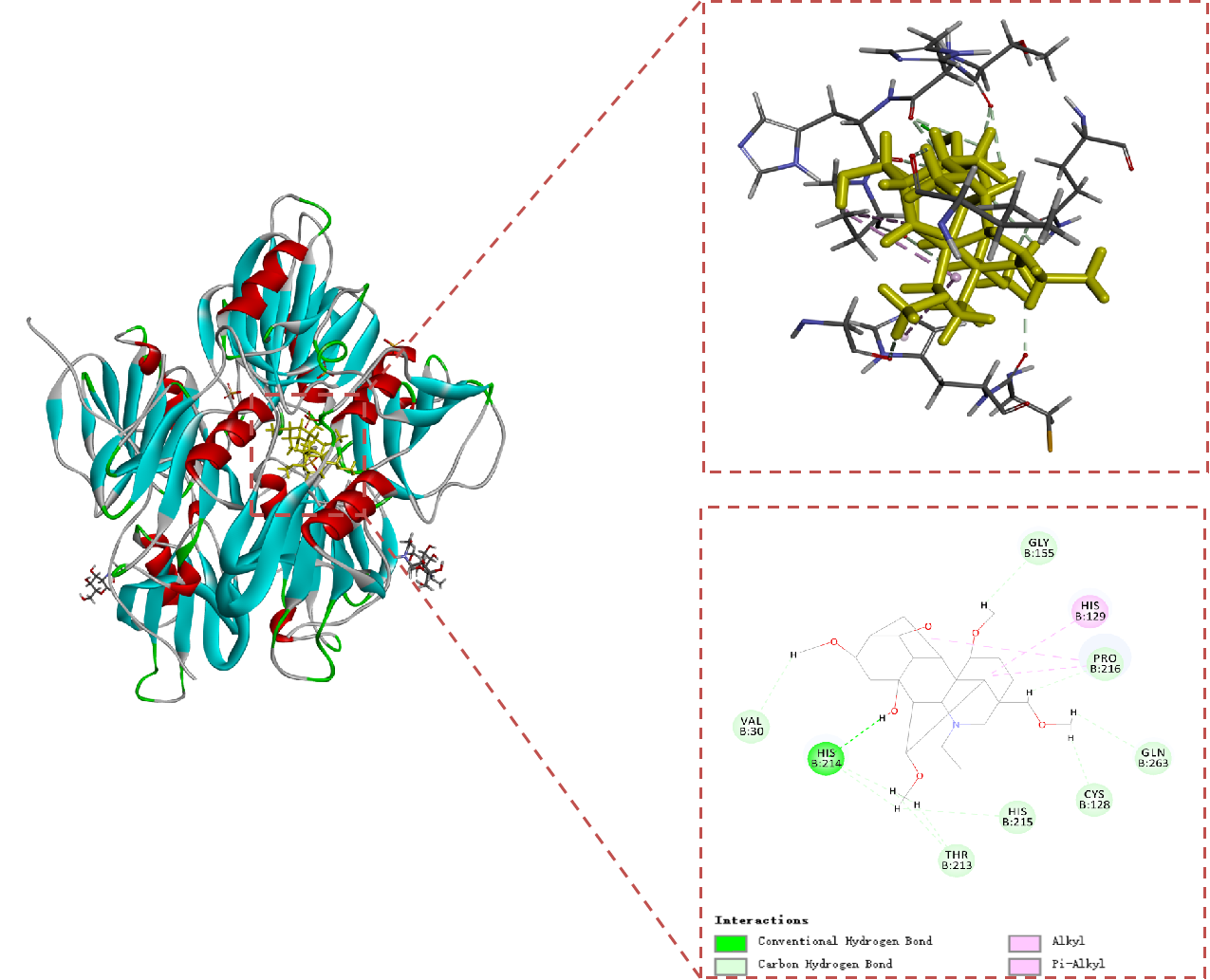


3.The 2D and 3D interaction diagrams of benzoylaconitine with the Sod1 protein


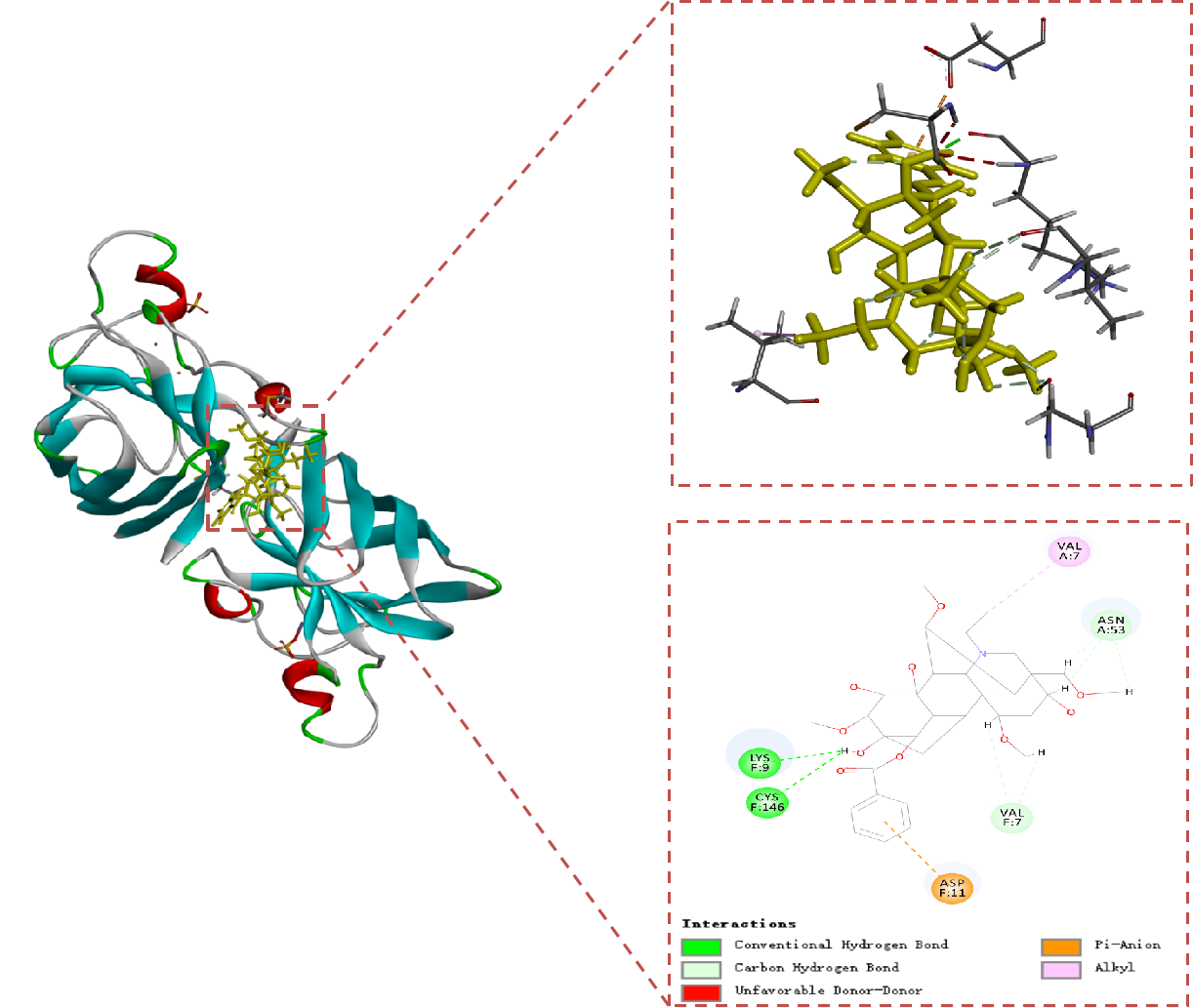


4.The 2D and 3D interaction diagrams of aconitine with the Casp3 protein.


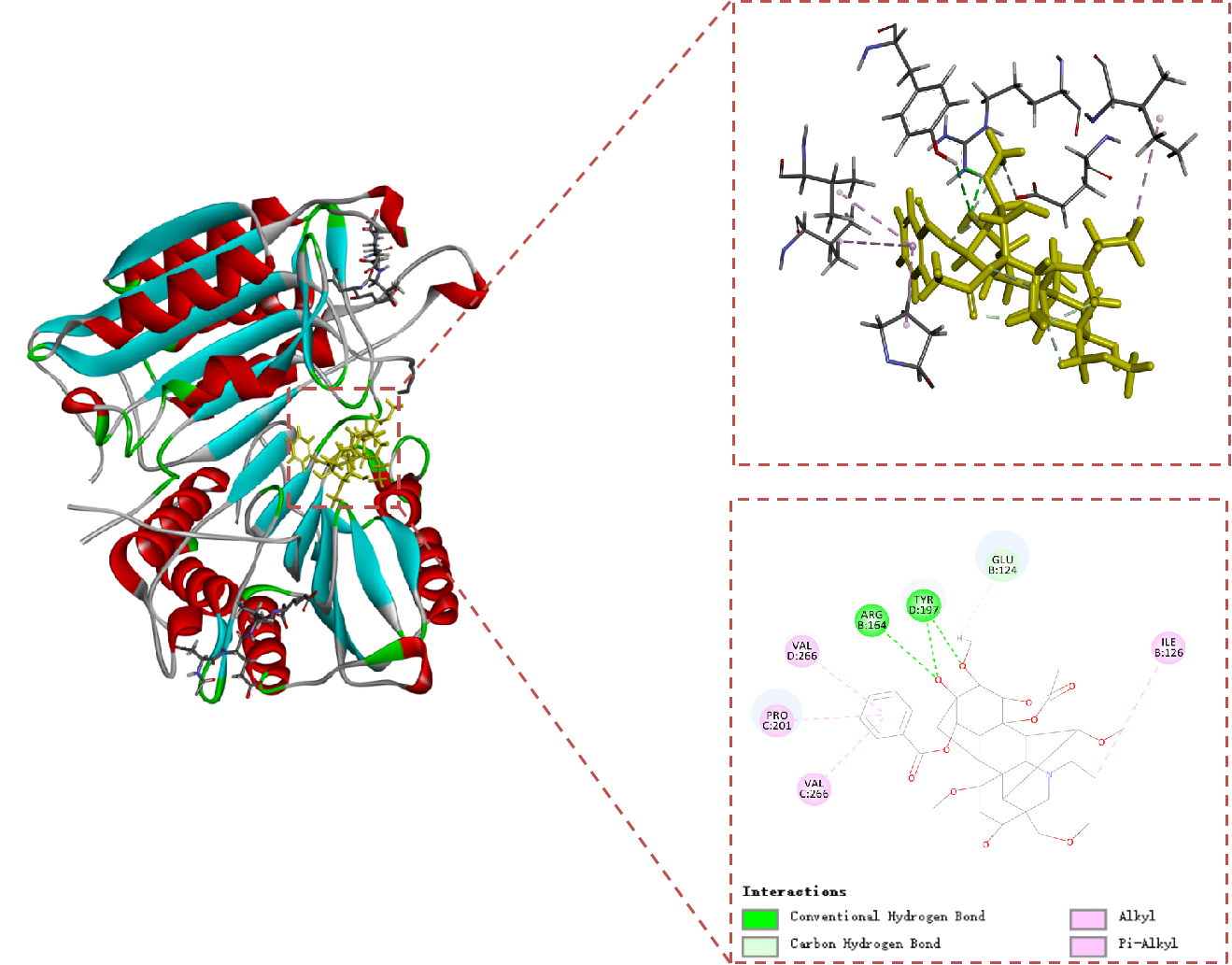


5.The 2D and 3D interaction diagrams of Senbusine A with the Bax protein.


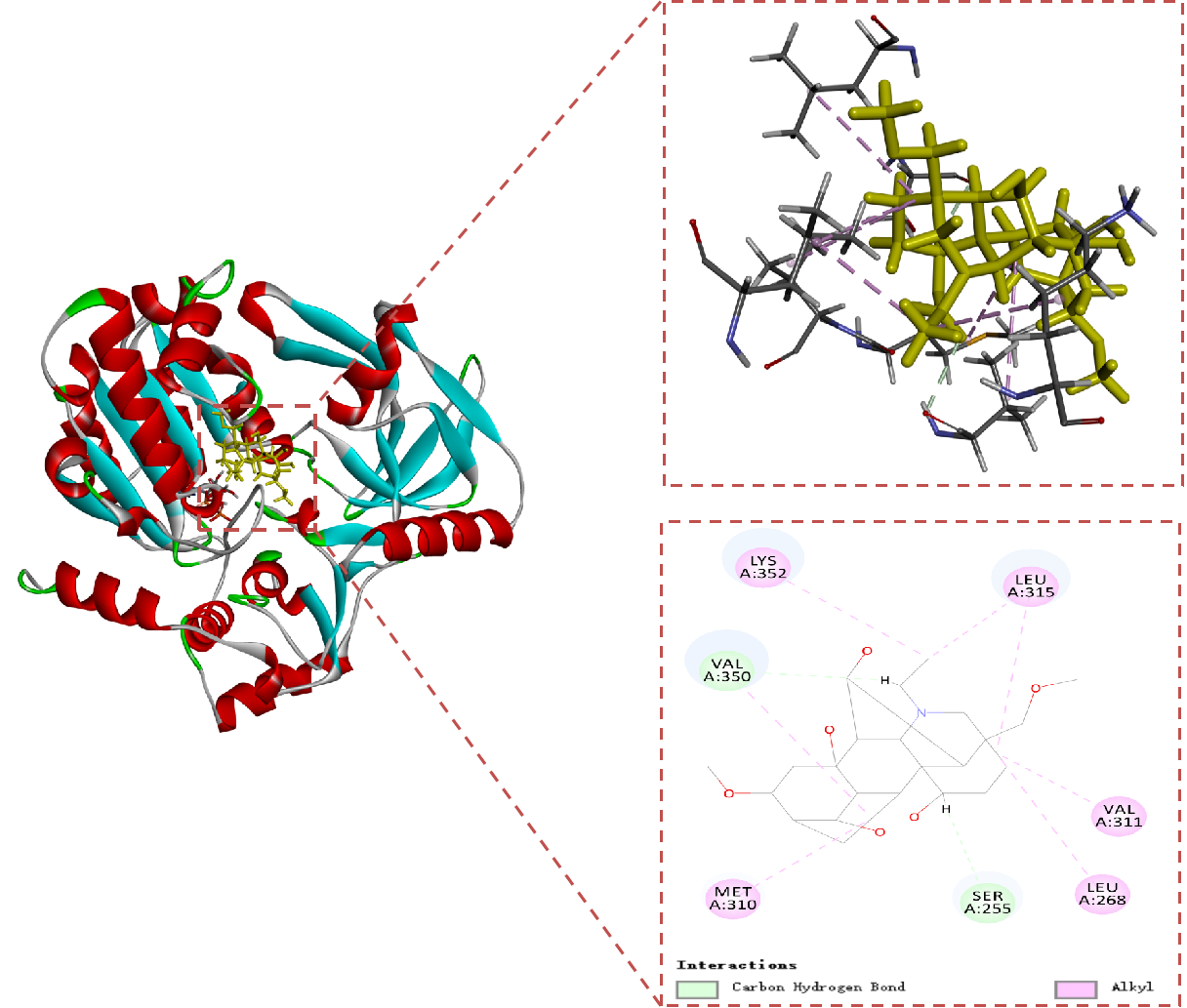


6.The 2D and 3D interaction diagrams of mesaconine with the Bcl2


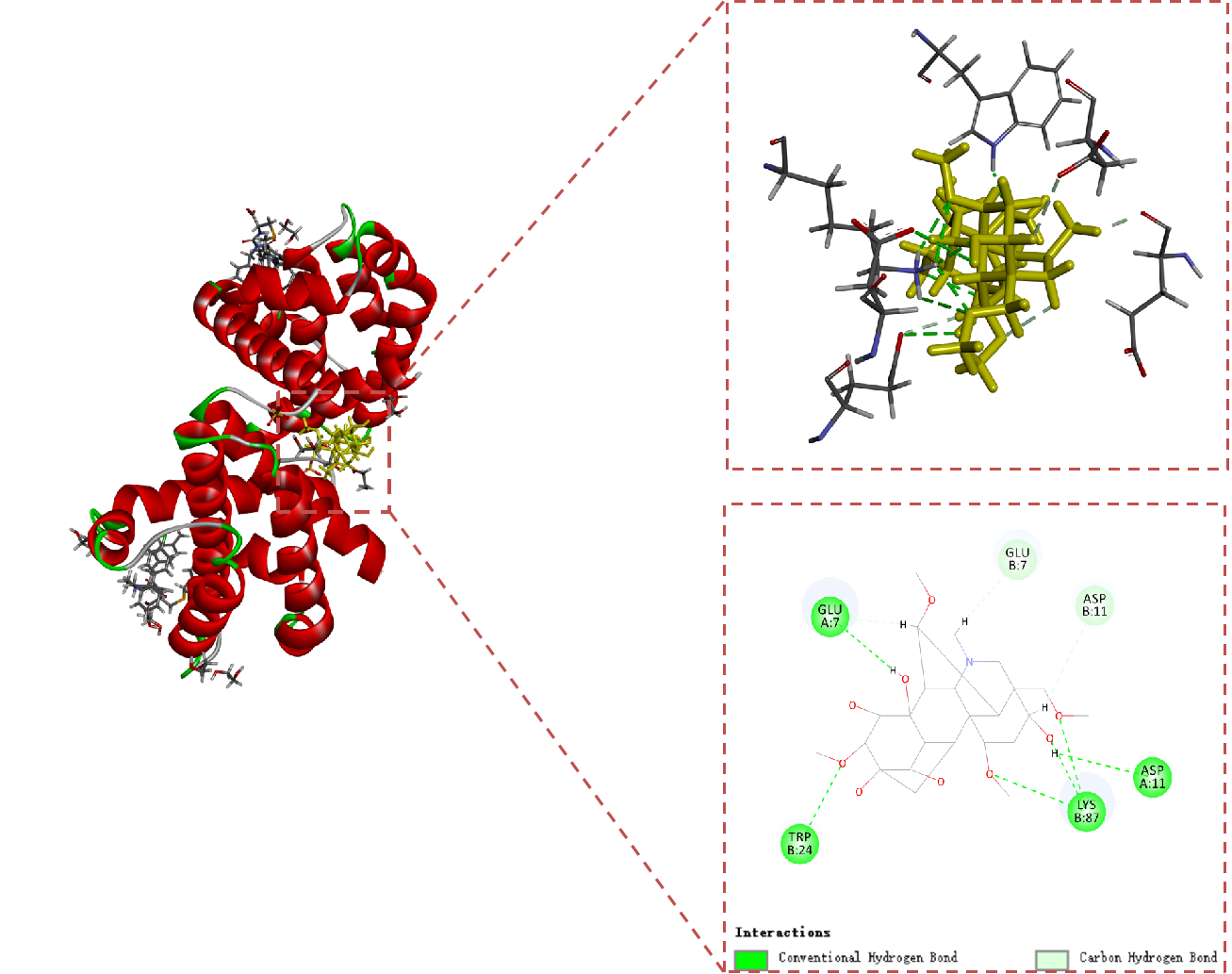


7.The 2D and 3D interaction diagrams of benzoylaconitine with the Ctsk protein.


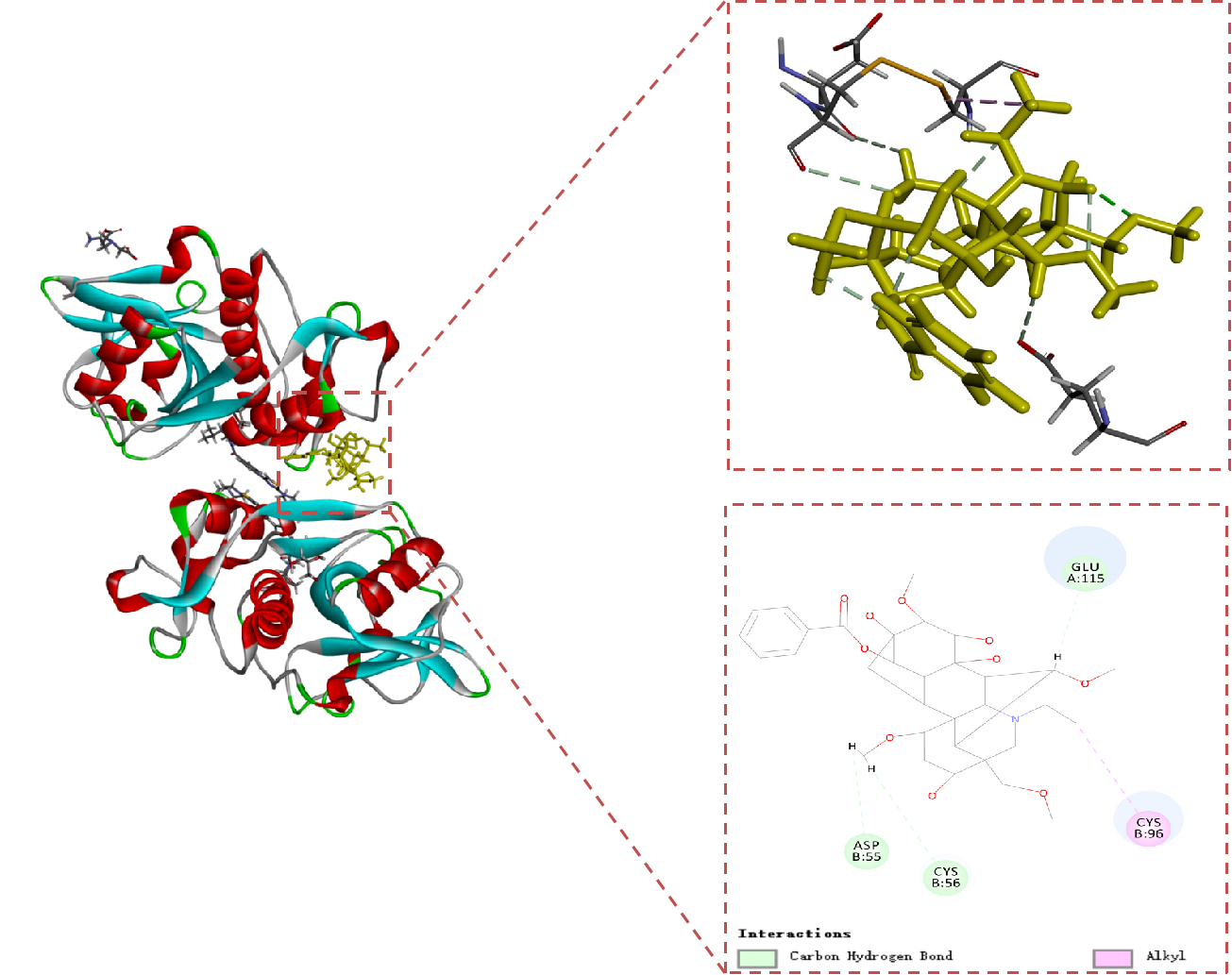


8.The 2D and 3D interaction diagrams of ellagic acid with the Acp5 protein.


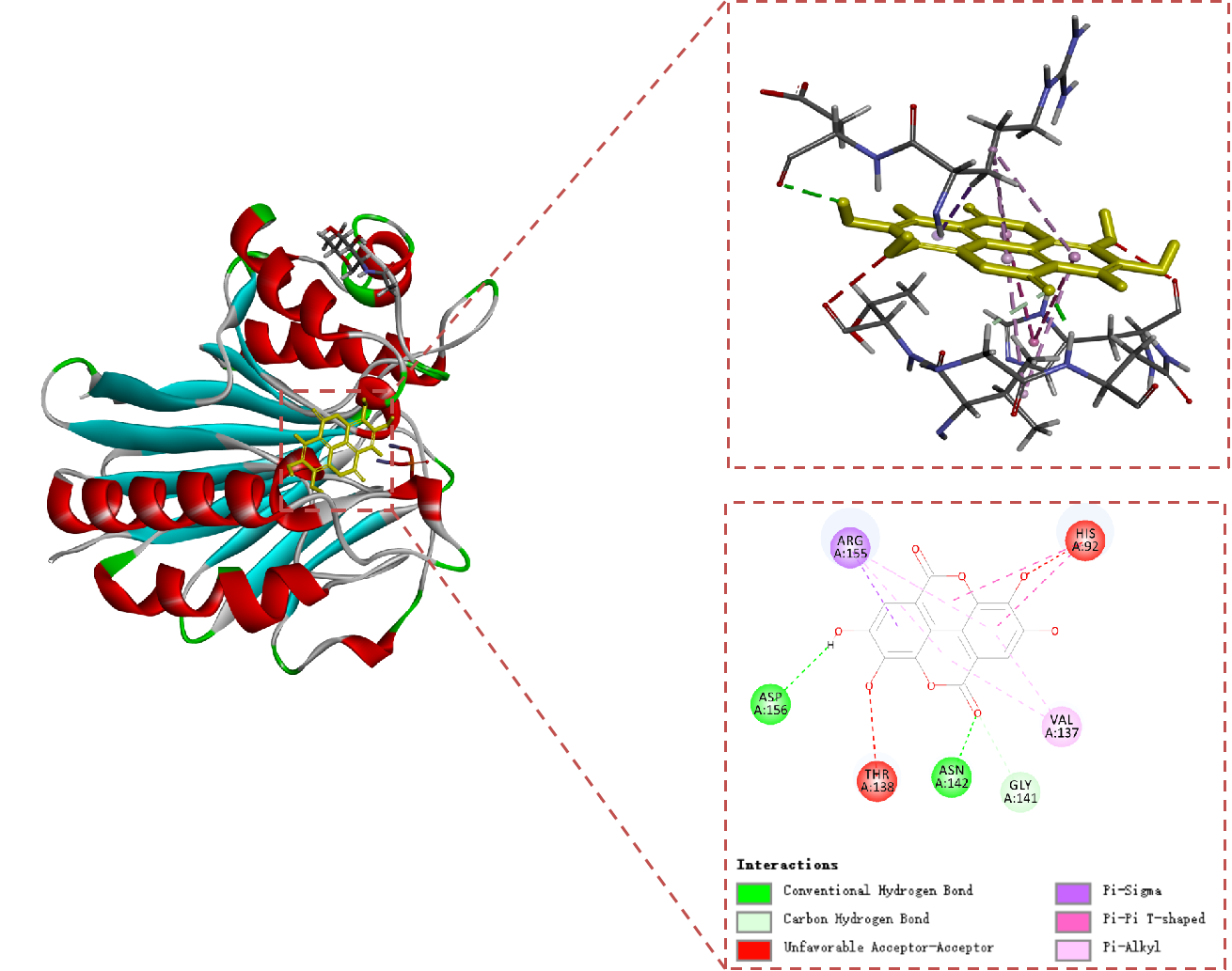

Supplement: Supplementary file 1 [file 13020_2025_1306_MOESM1_ESM.docx]
